# A Facile One-Pot Process for the Formation of Hindered Tertiary Amines 

**DOI:** 10.3390/molecules17055151

**Published:** 2012-05-03

**Authors:** Zhouyu Wang, Dong Pei, Yu Zhang, Chao Wang, Jian Sun

**Affiliations:** 1 Department of Pharmaceutics Engineering, Xihua University, Chengdu 610039, China; 2 Natural Products Research Center, Chengdu Institute of Biology, Chinese Academy of Sciences, Chengdu 610041, China

**Keywords:** reductive amination, tertiary amine, Lewis base, trichlorosilane, ketone

## Abstract

A simple and convenient method was developed for the preparation of hindered tertiary amines via direct reductive amination of ketones with secondary aryl amines, using trichlorosilane as reducing agent and tetramethylethylenediamine (TMEDA) as organic Lewis base activator. A broad range of ketones were reacted with *N*-methylaniline to afford the corresponding tertiary amine products in high yield. An open transition model was proposed for the reductive step.

## 1. Introduction

**Scheme 1:**
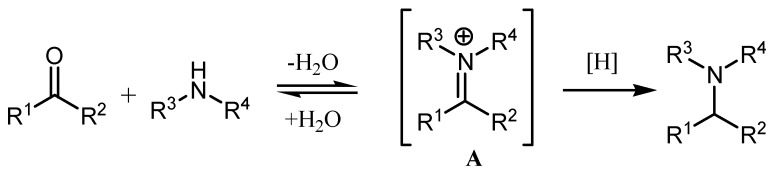
Direct reductive amination.

Tertiary amines are a key structural motif in numerous biologically active natural products such as alkaloids and pharmaceutical molecules. They is also regarded as one of the privileged functional groups in identifying lead compounds for drug discovery. Direct reductive amination (DRA) of carbonyls with amines (Scheme 1) is one of the most convenient and straightforward methods for the synthesis of amines [1,2,3,4,5,6,7,8,9,10,11,12,13]. It proceeds in a one-pot fashion under mild conditions and is compatible with many functional groups. While DRA has been widely used as a highly effective method for the preparation of primary and secondary amines, the application of such protocols for the synthesis of sterically demanding tertiary amines have been much less developed. This is particularly true for the preparation of sterically hindered tertiary arylamines.

In principle, both aldehydes and ketones are applicable in the DRA of secondary amines for the preparation of tertiary amines. In practice, aldehydes normally undergo smooth reactions and give good yields [14,15,16,17,18,19], whereas reactions with ketones have proven to be much more difficult due to increased steric hindrance. In the latter case, steric hindrance makes formation of the iminium intermediate **A** (Scheme 1) very difficult, and its formation is disfavored in the equilibrium, and inevitably the ketone is preferentially reduced with the reducing systems. In fact, successful and high-yielding intermolecular DRA approaches involving ketones and secondary amines have been limited to the reactions with less bulky cyclic ketones and/or less bulky cyclic amines [1,2,3,4,5,6,7,8,9,10,11,12,13]. Successful examples of acyclic ketone-participating intermolecular DRA with acyclic secondary amines are extremely rare [14,15,16,17]. Sodium cyanoborohydride (NaBH_3_CN) has been previously shown to be able to accomplish this transformation, but only gives poor yields. Recently, Xiao presented a highly effective new hydrogen transfer reduction system using an iridium complex as the catalyst, which was shown to efficiently promote the DRA of acyclic ketones with secondary amines [19]. However, the ketones that reacted with secondary amines with high efficiency are limited to aliphatic ones, aromatic ketones exhibited low reactivity and afforded low yields, with only one exception in that high yields could be achieved when the secondary amine partner is the cyclic amine pyrrolidine. Therefore, the development of a general and highly effective method for the intermolecular DRA of ketones with secondary amines represents a big challenge. Herein, we report that a metal-free reduction system could efficiently furnish the DRA of *N*-methylaniline with a broad range of ketones under mild conditions to produce sterically hindered tertiary amines in good to high yields.

Kobayashi first demonstrated that trichlorosilane and Lewis basic *N*, *N*-dimethylformamide (DMF) could form an efficient reduction system for the reductive amination of primary amines with aldehydes [20]. Later, we and others proved that use of chiral organic Lewis bases could enable this reduction system to be applicable for catalytic asymmetric indirect reductive amination (IRA) of prochiral ketones with primary amines [21,22,23,24,25,26,27,28,29,30,31,32,33,34,35,36,37,38,39,40,41,42,43], and high yields and excellent enantioselectivities have been obtained for a broad range of substrates using elaborate chiral Lewis base catalysts. We were interested to test if this reduction system could be applicable for the challenging intermolecular DRA of ketones and secondary amines, particularly the DRA of acyclic ketones and secondary amines.

## 2. Results and Discussion

Initially, we examined the DRA of acetophenone (**1a**) with different secondary amines using one equivalent of DMF as the Lewis base activator in dichloromethane at room temperature, and found that only the aromatic secondary arylamine **2** is reactive [44], which afforded the desired aromatic tertiary amine **3a** with 63% yield in 36 h (entry 2, Table 1). Next, we tested different organic Lewis bases as activator for the DRA of **1a** with **2**. As shown in Table 1, besides DMF and its analogue DMAc, other Lewis bases such as pyridine, 2,6-lutidine, diisopropylamine, triethylamine, and HMPA also promoted the reaction and afforded moderate to good yields (entries 4–8). Tetramethylethylenediamine (TMEDA) proved to be the best activator, furnishing product **3a** in up to 92% yield (entry 9).

**Table 1:** Direct reductive amination under different conditions ^a^. 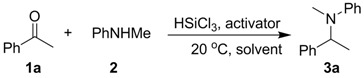

Entry	Solvent	Activator (equiv.)	Yield (%) *^b^*
1	DCM	-	19
2	DCM	DMF (1.0)	63
3	DCM	DMAc (1.0)	70
4	DCM	Lutidine (1.0)	78
5	DCM	Pyridine (1.0)	88
6	DCM	DIEA (1.0)	84
7	DCM	TEA (1.0)	43
8	DCM	HMPA (1.0)	68
9	DCM	TMEDA (1.0)	92
10	DCM	TMEDA (0.5)	85
11	DCM	TMEDA (0.2)	80
12	Toluene	TMEDA (1.0)	60
13	CH_3_CN	TMEDA (1.0)	76
14	CHCl_3_	TMEDA (1.0)	74
15	ClCH_2_CH_2_Cl	TMEDA (1.0)	73
16	THF	TMEDA (1.0)	24

*^a^* The reaction was carried out on a 0.2 mmol scale (**1a**:**2**:HSiCl_3_ = 1:1.2:2) in 1 mL solvent at room temperature for 36 h; *^b^* Isolated yield based on the ketone.

When the amount of TMEDA was decreased from 1.0 to 0.5 and 0.2 equivalent, the DRA of **1a** with **2** could also proceed to give **3a** with reasonable yields (85% and 80%, respectively, entries 10 and 11), implying that this transformation could be catalytic.

The TMEDA-promoted DRA of **1a** with **2** could also be run in different solvents other than dichloromethane, including toluene, acetonitrile, chloroform, and dichloroethane, albeit with moderate yields (entries 12–16).

To probe the generality of the TMEDA-promoted DRA by trichlorosilane, we next examined various ketones as substrates under optimal conditions. Table 2 summarizes the results. The aromatic ketones, either electron-deficient or electron-rich, all underwent facile DRA with **2** to afford the desired tertiary amine products in good to high yields (entries 1–14). In particular, the *ortho*-substituted phenylmethyl ketones **1m** and **1n**, which are normally more difficult substrates due to steric hindrance and/or electronic interference, also proved to be good substrates (entries 13 and 14). The α,β-unsaturated ketone **1o** was also found to be an excellent substrate, which gave the desired 1,2-reduction amine product with up to 90% yield (entry 15). The possible 1,4-reduction and Michael addition by-products were not observed. Moreover, the DRA of cyclic ketone **1p** and acyclic alipahtic ketones **1q** and **1r** could also be accomplished to afford the corresponding amine products in good to high yields (entries 16–18).

**Table 2:** Direct reductive amination of various acetophenone analogues with *N*-methylaniline *^a^*. 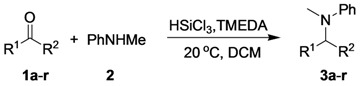

Entry	Ketone	Yield (%) *^b^*	Entry	Ketone	Yield (%) *^b^*
1	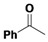 **(1a)**	92	10	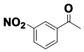 **(1j)**	62
2	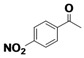 **(1b)**	76	11	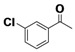 **(1k)**	82
3	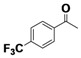 **(1c)**	83	12	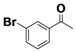 **(1l)**	89
4	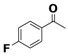 **(1d)**	86	13	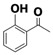 **(1m)**	88
5	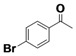 **(1e)**	85	14	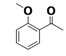 **(1n)**	61
6	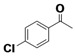 **(1f)**	89	15	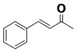 **(1o)**	90
7	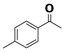 **(1g)**	60	16	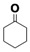 **(1p)**	88
8	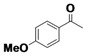 **(1h)**	87	17	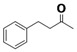 **(1q)**	76
9	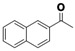 **(1i)**	79	18	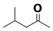 **(1r)**	66

*^a^* The reaction was carried out on a 0.2 mmol scale (**1**:**2**:TMEDA:HSiCl_3_ = 1:1.2:1:2) in 1 mL solvent at room temperature for 36 h; *^b^* Isolated yield based on the ketone.

**Scheme 2:**
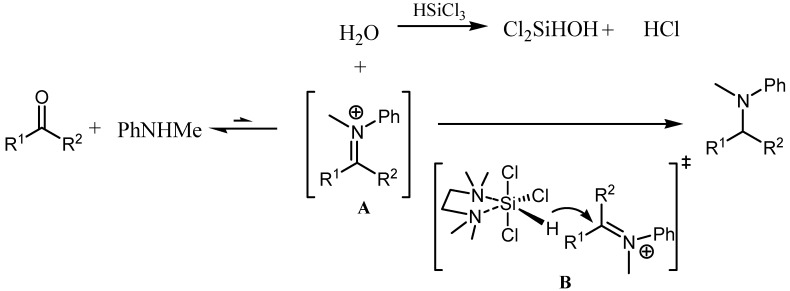
A plausible mechanism for the TMEDA-promoted direct reductive amination of ketone with secondary arylamine.

In Scheme 2, a plausible reaction mechanism was proposed for the present Lewis base-promoted DRA of ketones with *N*-methylaniline. The ketone reacts with the amine first to yield iminium intermediate **A** and release one equivalent of water. Supposedly, this step is very difficult and slow due to steric hindrance and the equilibrium is much more favourable for the backwards conversion, which is also commonly complicated with the reduction of the ketone. The reasons why the present reduction system could be highly effective most likely lie in the following facts: the reductant HSiCl_3_ also happens to be a good water scavenger, which helps to push the iminium ion formation step forwards; moreover, the Lewis base-HSiCl_3_ reduction system is known to have much lower reactivity towards C=O bond than C=N bond [23], which largely avoids the unwanted reduction of ketone.

The reduction of the imine intermediate **A** by the TMEDA-activated HSiCl_3_ is proposed to pass through transition state **B**. Unlike the previously proposed transition models for the reduction of primary amine derived imines that feature a closed structure due to the interaction of the imine nitrogen with either the central silicon or the hydrogen donor(s) of the catalyst for activation [23,24], **B** has a fully occupied iminium nitrogen and can only adopt an open structure.

## 3. Experimental

### 3.1. General

All starting materials were of the highest commercially available grade and used without further purification. All solvents used in the reactions were distilled from appropriate drying agents prior to use. Reactions were monitored by thin layer chromatography using silica gel HSGF254 plates. Flash chromatography was performed using silica gel HG/T2354-92. ^1^H - and ^13^C-NMR (300 or 600 and 75 or 150 MHz, respectively) spectra were recorded in CDCl_3_. ^1^H-NMR chemical shifts are reported in ppm (**δ**) relative to tetramethylsilane (TMS) with the solvent resonance employed as the internal standard (CDCl_3_, **δ** 7.26 ppm). Data are reported as follows: chemical shift, multiplicity (s = singlet, d = doublet, t = triplet, m = multiplet), coupling constants (Hz) and integration. ^13^C-NMR chemical shifts are reported in ppm from tetramethylsilane (TMS) with the solvent resonance as the internal standard (CDCl_3_, **δ** 77.0 ppm). ESIMS spectra were recorded on BioTOF Q. Chemical yields refer to pure isolated substances.

### 3.2. General Procedure for the Direct Reductive Amination of Ketones with N-Methylaniline

To a solution of ketone (0.2 mmol) and amine (0.24 mmol) in dichloromethane (1.0 mL) was added tetramethylethylenediamine (TMEDA) (22 mg, 0.2 mmol). After stirring at room temperature for 0.5 h, trichlorosilane (40 μL, 0.4 mmol) was added, and the mixture was continued to stir for 36 h. The reaction mixture was then quenched with saturated aqueous sodium bicarbonate and was extracted with dichloromethane. The combined extracts were washed with water, dried over MgSO_4_, and concentrated under vacuum. The residue was purified by column chromatography over silica gel (hexane/ethyl acetate as eluent) to afford pure product.

*N-Methyl-N-(1-phenylethyl)aniline* (**3a**). Light yellow oil; Yield: 92%; purification by flash chromatography (hexane/EtOAc = 20:1). ^1^H-NMR (300 MHz, CDCl_3_): δ = 1.57 (d, *J* = 6.90 Hz, 3H), 2.70 (s, 3H), 5.15 (q, *J* = 6.91 Hz, 1H), 6.75 (t, *J* = 7.24 Hz, 3H), 6.86 (d, *J* = 8.15 Hz, 2H), 7.25–7.38 (m, 7H); ^13^C-NMR (150 MHz, CDCl_3_): δ = 16.3, 31.9, 56.6, 113.1, 116.7, 126.8, 126.9, 128.4, 129.2, 142.8, 150.3; ESI HRMS exact mass calcd. for (C_1__5_H_17_N_1_ + H)^+^, requires *m/z* 212.1439, found *m/z* 212.1440.

*N-Methyl-N-(1-(4-nitrophenyl)ethyl)aniline* (**3b**). Light yellow oil; Yield: 76%; purification by flash chromatography (hexane/EtOAc = 20:1); ^1^H-NMR (300 MHz, CDCl_3_): δ = 1.61 (d, *J* = 6.91 Hz, 3H), 2.72 (s, 3H), 5.15 (q, *J* = 6.99 Hz, 1H), 6.77–6.81 (m, 3H), 7.26–7.29 (m, 2H), 7.49 (d, *J* = 8.51 Hz, 2H), 8.18 (d, *J* = 8.74 Hz, 2H). ^13^C-NMR (75 MHz, CDCl_3_): δ = 16.6, 32.3, 56.9, 113.5, 117.7, 123.7, 127.7, 129.3, 147.0, 149.7, 150.9; ESI HRMS exact mass calcd. for (C_1__5_H_1__6_N_2_O_2_ + H)^+^, requires *m/z* 257.1285, found *m/z* 257.1281.

*N-Methyl-N-(1-(4-(trifluoromethyl)phenyl)ethyl)aniline* (**3c**). Light yellow oil; Yield: 83%; purification by flash chromatography (hexane/EtOAc = 20:1); ^1^H-NMR (300 MHz, CDCl_3_): δ = 1.58 (d, *J* = 6.91 Hz, 3H), 2.71 (s, 3H), 5.14 (q, *J* = 6.85 Hz, 1H), 6.75–6.80 (m, 1H), 6.84 (d, *J* = 8.02 Hz, 2H), 7.24–7.30 (m, 2H), 7.44 (d, *J* = 8.57 Hz, 2H), 7.60 (d, *J* = 8.22 Hz, 2H). ^13^C-NMR (75 MHz, CDCl_3_): δ = 16.5, 32.0, 56.7, 113.4, 117.3, 125.4, 126.8, 129.3, 147.2, 150.0; ESI HRMS exact mass calcd. for (C_1__6_H_1__6_F_3_N_1_ + H)^+^, requires *m/z* 280.1308, found *m/z* 280.1294.

*N-(1-(4-Fluorophenyl)ethyl)-N-methylaniline* (**3d**). Light yellow oil; Yield: 86%; purification by flash chromatography (hexane/EtOAc = 20:1); ^1^H-NMR (300 MHz, CDCl_3_): δ = 1.53 (d, *J* = 6.93 Hz, 3H), 2.66 (s, 3H), 3.81 (s, 3H), 5.11 (q, *J* = 6.84 Hz, 1H), 6.74 (t, *J* = 7.12 Hz, 1H), 6.86 (d, *J* = 8.37 Hz, 2H), 7.04 (t,*J* = 8.69 Hz, 2H), 7.23–7.29 (m, 4H). ^13^C-NMR (75 MHz, CDCl_3_): δ = 16.4, 31.8, 56.2, 113.4, 115.0, 117.0, 128.4, 129.2, 138.5, 150.2, 161.8 (d, *J* = 243 Hz); ESI HRMS exact mass calcd. for (C_1__5_H_1__6_F_1_N_1_ + H)^+^, requires *m/z* 230.1340, found *m/z* 230.1341.

*N-(1-(4-Bromophenyl)ethyl)-N-methylaniline* (**3e**). Light yellow oil; Yield: 85%; purification by flash chromatography (hexane/EtOAc = 20:1); ^1^H-NMR (300 MHz, CDCl_3_): δ = 1.53 (d, *J* = 6.88 Hz, 3H), 2.67 (s, 3H), 5.06 (q, *J* = 6.92 Hz, 1H), 6.74 (t, *J* = 7.21 Hz, 1H), 6.83 (d, *J* = 8.19 Hz, 2H),7.22–7.28 (m, 4H), 7.46 (d, *J* = 6.72 Hz, 2H). ^13^C-NMR (75 MHz, CDCl_3_): δ = 16.3, 31.8, 56.2, 113.3, 117.0, 120.7, 128.7, 129.2, 131.4, 141.9, 150.0; ESI HRMS exact mass calcd. for (C_1__5_H_1__6_Br_1_N_1_ + H)^+^, requires *m/z* 290.0539, found *m/z* 290.0535.

*N-(1-(4-Chlorophenyl)ethyl)-N-methylaniline* (**3f**). Light yellow oil; Yield: 89%; purification by flash chromatography (hexane/EtOAc = 20:1); ^1^H-NMR (300 MHz, CDCl_3_): δ = 1.55 (d, *J* = 6.87 Hz, 3H), 2.68 (s, 3H), 5.10 (q, *J* = 6.88 Hz, 1H), 6.76 (t, *J* = 7.25 Hz, 1H), 6.84 (d, *J* = 8.13 Hz, 2H),7.24–7.32 (m, 6H). ^13^C-NMR (75 MHz, CDCl_3_): δ = 16.3, 31.8, 56.2, 113.3, 117.0, 128.3, 128.5, 129.2, 132.5, 141.4, 150.0; ESI HRMS exact mass calcd. for (C_1__5_H_1__6_Cl_1_N_1_ + H)^+^, requires *m/z* 246.1044, found *m/z* 246.1038.

*N-Methyl-N-(1-p-tolylethyl)aniline* (**3g**). Light yellow oil; Yield: 60%; purification by flash chromatography (hexane/EtOAc = 20:1); ^1^H-NMR (300 MHz, CDCl_3_): δ = 1.55 (d, *J* = 6.88 Hz, 3H), 2.36 (s, 3H), 2.69 (s, 3H), 5.12 (q, *J* = 6.89 Hz, 1H), 6.74 (t, *J* = 7.28 Hz, 1H), 6.84 (d, *J* = 8.28 Hz, 2H), 7.14–7.29 (m, 6H). ^13^C-NMR (75 MHz, CDCl_3_): δ = 16.3, 21.0, 31.8, 56.4, 113.2, 116.7, 126.9, 129.0, 129.1, 136.4, 139.6, 150.1; ESI HRMS exact mass calcd. for (C_1__6_H_1__9_N_1_ + H)^+^, requires *m/z* 226.1590, found *m/z* 226.1592.

*N-(1-(4-Methoxyphenyl)ethyl)-N-methylaniline* (**3h**). Light yellow oil; Yield: 87%; purification by flash chromatography (hexane/EtOAc = 20:1); ^1^H-NMR (300 MHz, CDCl_3_): δ = 1.53 (d, *J* = 6.87 Hz, 3H), 2.66 (s, 3H), 3.82 (s, 3H), 5.11 (q, *J* = 6.66 Hz, 1H), 6.74 (t, *J* = 7.23 Hz, 1H), 6.84–6.90 (m, 4H),7.23–7.29 (m, 4H). ^13^C-NMR (75 MHz, CDCl_3_): δ = 16.1, 31.6, 55.2, 55.9, 113.1, 113.6, 116.5, 128.0, 129.1, 134.7, 150.2, 158.4; ESI HRMS exact mass calcd. for (C_1__6_H_1__9_N_1_O_1_ + H)^+^, requires *m/z* 242.1539, found *m/z* 242.1547.

*N-Methyl-N-(1-(naphthalen-2-yl)ethyl)aniline* (**3i**). Light yellow oil; Yield: 79%; purification by flash chromatography (hexane/EtOAc = 20:1); ^1^H-NMR (300 MHz, CDCl_3_): δ = 1.67 (d, *J* = 6.84 Hz, 3H), 2.72 (s, 3H), 5.30 (q, *J* = 6.74 Hz, 1H), 6.78 (t, *J* = 8.31 Hz, 1H), 6.92 (d, *J* = 8.31 Hz, 2H). 7.26–7.32 (m, 2H), 7.46–7.51 (m, 3H), 7.77–7.86 (m, 4H); ^13^C-NMR (75 MHz, CDCl_3_): δ = 16.0, 31.9, 56.7, 113.2, 116.8, 124.9, 125.7, 126.0, 127.6, 127.9, 128.1, 129.3, 132.6, 133.4, 140.5, 150.2; ESI HRMS exact mass calcd. for (C_1__9_H_1__9_N_1_ + H)^+^, requires *m/z* 262.1590, found *m/z* 262.1596.

*N-Methyl-N-(1-(3-nitrophenyl)ethyl)aniline* (**3j**). Light yellow oil; Yield: 62%; purification by flash chromatography (hexane/EtOAc = 20:1); ^1^H-NMR (300 MHz, CDCl_3_): δ = 1.61 (d, *J* = 6.90 Hz, 3H), 2.71 (s, 3H), 5.16 (q, *J* = 6.76 Hz, 1H), 6.77–6.87 (m, 3H), 7.26–7.30 (m, 2H), 7.50 (t, *J* = 7.91 Hz, 1H), 7.66 (d, *J* = 7.73 Hz, 1H), 8.12 (d, *J* = 8.06 Hz, 1H), 8.21 (s, 1H). ^13^C-NMR (75 MHz, CDCl_3_): δ = 16.3, 32.0, 56.7, 113.6, 117.7, 121.7, 122.1, 129.3, 133.2, 145.5, 148.6, 149.8; ESI HRMS exact mass calcd. for (C_1__5_H_1__6_O_2_N_2_ + H)^+^, requires *m/z* 257.1285, found *m/z* 257.1283.

*N-(1-(3-Chlorophenyl)ethyl)-N-methylaniline* (**3k**). Light yellow oil; Yield: 82%; purification by flash chromatography (hexane/EtOAc = 20:1); ^1^H-NMR (300 MHz, CDCl_3_): δ = 1.55 (d, *J* = 6.89 Hz, 3H), 2.71 (s, 3H), 5.14 (q, *J* = 6.88 Hz, 1H), 6.78 (t, *J* = 7.26 Hz, 1H), 6.83 (d, *J* = 8.10 Hz, 2H), 7.20–7.35 (m, 6H); ^13^C-NMR (75 MHz, CDCl_3_): δ = 16.3, 32.0, 56.5, 113.3, 117.1, 125.1, 127.0, 129.2, 129.6, 134.4, 145.2, 150.0; ESI HRMS exact mass calcd. for (C_1__5_H_1__6_ClN + H)^+^, requires *m/z* 246.1044, found *m/z* 246.1034.

*N-(1-(3-Bromophenyl)ethyl)-N-methylaniline* (**3l**). Light yellow oil; Yield: 89%; purification by flash chromatography (hexane/EtOAc = 20:1); ^1^H-NMR (300 MHz, CDCl_3_): δ = 1.55 (d, *J* = 6.89 Hz, 3H), 2.71 (s, 3H), 5.10 (q, *J* = 6.89 Hz, 1H), 6.78 (t, *J* = 7.28 Hz, 1H), 6.85 (d, *J* = 8.23 Hz, 2H), 7.18–7.31 (m, 4H), 7.42 (d, *J* = 7.52 Hz, 1H), 7.50 (s, 1H); ^13^C-NMR (75 MHz, CDCl_3_): δ = 16.3, 32.0, 56.5, 113.3, 117.1, 122.7, 124.9, 125.5, 129.2, 129.9, 145.5, 150.0; ESI HRMS exact mass calcd. for (C_1__5_H_1__6_BrN + H)^+^, requires *m/z* 290.0539, found *m/z* 290.0544.

*2-(1-(Methyl(phenyl)amino)ethyl)phenol* (**3m**). Light yellow oil; Yield: 88%; purification by flash chromatography (hexane/EtOAc = 20:1); ^1^H-NMR (300 MHz, CDCl_3_): δ = 1.35 (d, *J* = 6.75 Hz, 3H), 2.63 (s, 3H), 4.93 (q, *J* = 6.69 Hz, 1H), 6.87–6.93 (m, 2H), 7.05–7.16 (m, 2H), 7.21–7.25 (m, 3H), 7.34–7.39 (m, 2H), 10.08 (s, 1H); ^13^C-NMR (75 MHz, CDCl_3_): δ = 12.9, 34.3, 60.3, 116.4, 119.6, 120.0, 122.7, 126.3, 127.1, 128.9, 129.3, 149.1, 157.2; ESI HRMS exact mass calcd. for (C_1__5_H_1__7_ON + H)^+^, requires *m/z* 228.1383, found *m/z* 228.1387.

*N-(1-(2-Methoxyphenyl)ethyl)-N-methylaniline* (**3n**). Light yellow oil; Yield: 61%; purification by flash chromatography (hexane/EtOAc = 20:1); ^1^H-NMR (300 MHz, CDCl_3_): δ = 1.50 (d, *J* = 6.98 Hz, 3H), 2.83 (s, 3H), 3.80 (s, 3H), 5.32 (q, *J* = 6.96 Hz, 1H), 6.67 (t, *J* = 7.21 Hz, 1H), 6.81 (d, *J* = 8.08 Hz, 2H), 6.87–6.93 (m, 2H), 7.17–7.26 (m, 4H); ^13^C-NMR (75 MHz, CDCl_3_): δ = 16.9, 31.9, 52.3, 55.4, 110.7, 113.2, 116.3, 120.3, 127.0, 128.0, 128.8, 131.6, 150.2, 157.3; ESI HRMS exact mass calcd. for (C_1__6_H_1__9_NO + H)^+^, requires *m/z* 242.1539, found *m/z* 242.1554.

*(E)-N-Methyl-N-(4-phenylbut-3-en-2-yl)aniline* (**3o**). Light yellow oil; Yield: 90%; purification by flash chromatography (hexane/EtOAc = 20:1); ^1^H-NMR (300 MHz, CDCl_3_): δ = 1.37 (d, *J* = 6.78 Hz, 3H), 2.80 (s, 3H), 4.66 (m, 1H), 6.32 (dd, *J* = 16.15, 4.37 Hz, 1H), 6.47 (d, *J* = 16.15 Hz, 1H), 6.74 (m, 1H), 6.85 (d, *J* = 8.01 Hz, 2H), 7.23–7.40 (m, 7H); ^13^C-NMR (75 MHz, CDCl_3_): δ = 16.2, 31.7, 54.9, 113.4, 116.8, 126.3, 127.4, 128.5, 129.1, 130.0, 131.5, 137.0, 150; ESI HRMS exact mass calcd. for (C_1__7_H_1__9_N + H)^+^, requires *m/z* 238.1590, found *m/z* 238.1598.

*N-Cyclohexyl-N-methylaniline* (**3p**). Light yellow oil; Yield: 88%; purification by flash chromatography (hexane/EtOAc = 20:1); ^1^H-NMR (300 MHz, CDCl_3_): δ = 1.12–1.44 (m, 5H), 1.67–1.87 (m, 5H), 2.78 (s, 3H), 3.54–3.61 (m, 1H), 6.69 (t, *J* = 7.22 Hz, 1H), 6.79 (d, *J* = 8.29 Hz, 2H), 7.24 (t, *J* = 7.56 Hz, 2H); ^13^C-NMR (75 MHz, CDCl_3_): δ = 26.0, 26.2, 30.0, 31.2, 58.2, 113.2, 116.2, 129.0; ESI HRMS exact mass calcd. for (C_1__3_H_1__9_N + H)^+^, requires *m/z* 190.1596, found *m/z* 190.1602.

*N-Methyl-N-(4-phenylbutan-2-yl)aniline* (**3q**). Light yellow oil; Yield: 76%; purification by flash chromatography (hexane/EtOAc = 20:1); ^1^H-NMR (300 MHz, CDCl_3_): δ = 1.18 (d, *J* = 6.60 Hz, 3H), 1.80–1.87 (m, 1H), 1.95–2.03 (m, 1H), 2.62–2.69 (m, 2H), 2.79 (s, 3H), 3.92–3.99 (m, 1H), 6.71–6.79 (m, 3H), 7.19–7.33 (m, 7H); ^13^C-NMR (75 MHz, CDCl_3_): δ = 16.9, 29.8, 33.1, 36.4, 52.7, 113.1, 116.3, 125.7, 128.3, 128.4, 129.1, 142.1, 150.5; ESI HRMS exact mass calcd. for (C_1__7_H_21_N + H)^+^, requires *m/z* 240.1747, found *m/z* 240.1738.

*N-Methyl-N-(4-methylpentan-2-yl)aniline* (**3r**). Light yellow oil; Yield: 66%; purification by flash chromatography (hexane/EtOAc = 20:1); ^1^H-NMR (300 MHz, CDCl_3_): δ = 0.88 (d, *J* = 6.34 Hz, 3H), 0.91 (d, *J* = 6.34 Hz, 3H), 1.08 (d, *J* = 6.55 Hz, 3H), 1.25–1.27 (m, 1H), 1.53–1.61 (m, 2H) 2.69 (s, 3H), 4.00 (m, 1H), 6.67 (t, *J* = 7.23 Hz, 1H), 6.78 (d, *J* = 8.26 Hz, 2H), 7.20–7.26 (m, 2H); ^13^C-NMR (75 MHz, CDCl_3_): δ = 17.0, 22.6, 23.0, 25.1, 29.8, 43.7, 50.9, 112.8, 116.0, 129.1, 150.5; ESI HRMS exact mass calcd. for (C_1__3_H_21_N + H)^+^, requires *m/z* 192.1752, found *m/z* 192.1750.

## 4. Conclusions

In summary, we have developed a practical protocol enabling the direct reductive amination of a broad range of ketones with a secondary arylamine using trichlorosilane as the reducing agent and TMEDA as the Lewis base activator. This protocol provides a facile and efficient method for the preparation of bulky tertiary amines under mild and metal-free conditions. The development of the asymmetric version of this protocol is underway.

## Supplementary Materials

Supplementary materials can be accessed at: http://www.mdpi.com/1420-3049/17/5/5151/s1.
