# Supplementary material for: A Facile One-Pot Process for the Formation of Hindered Tertiary Amines
Source: Molecules. 2012 May 3;17(5):5151–63. doi: 10.3390/molecules17055151 (PMC6268708; doi:10.3390/molecules17055151)

The-NMR spectra of **3a**

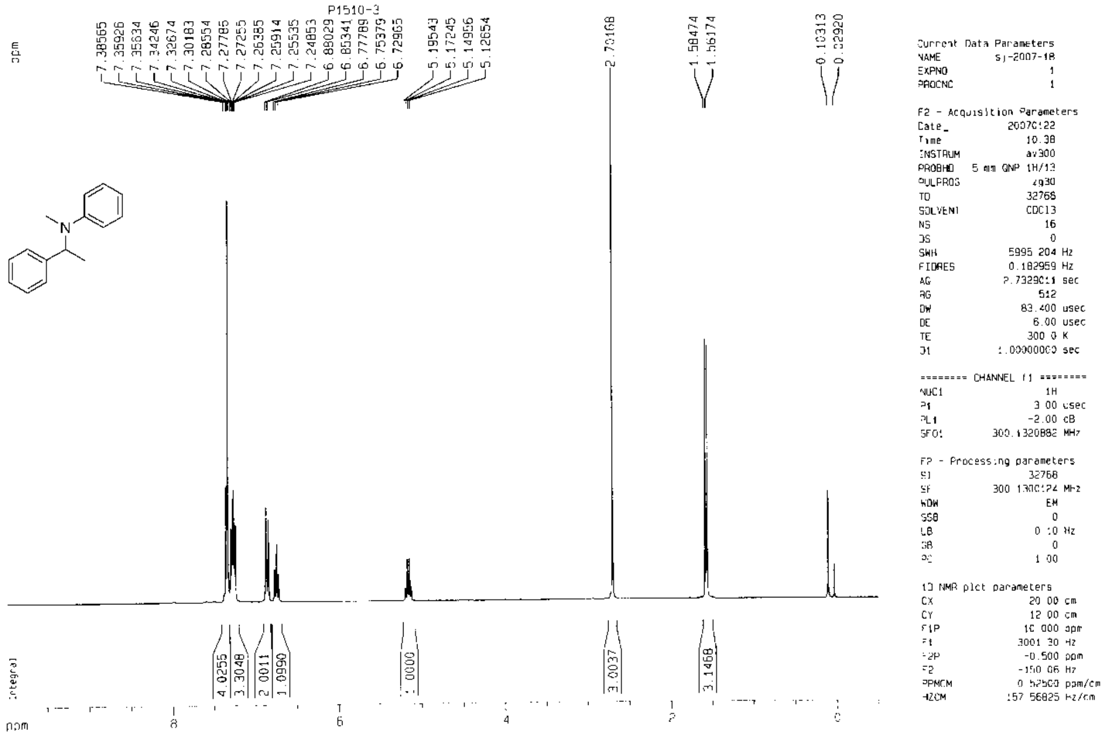

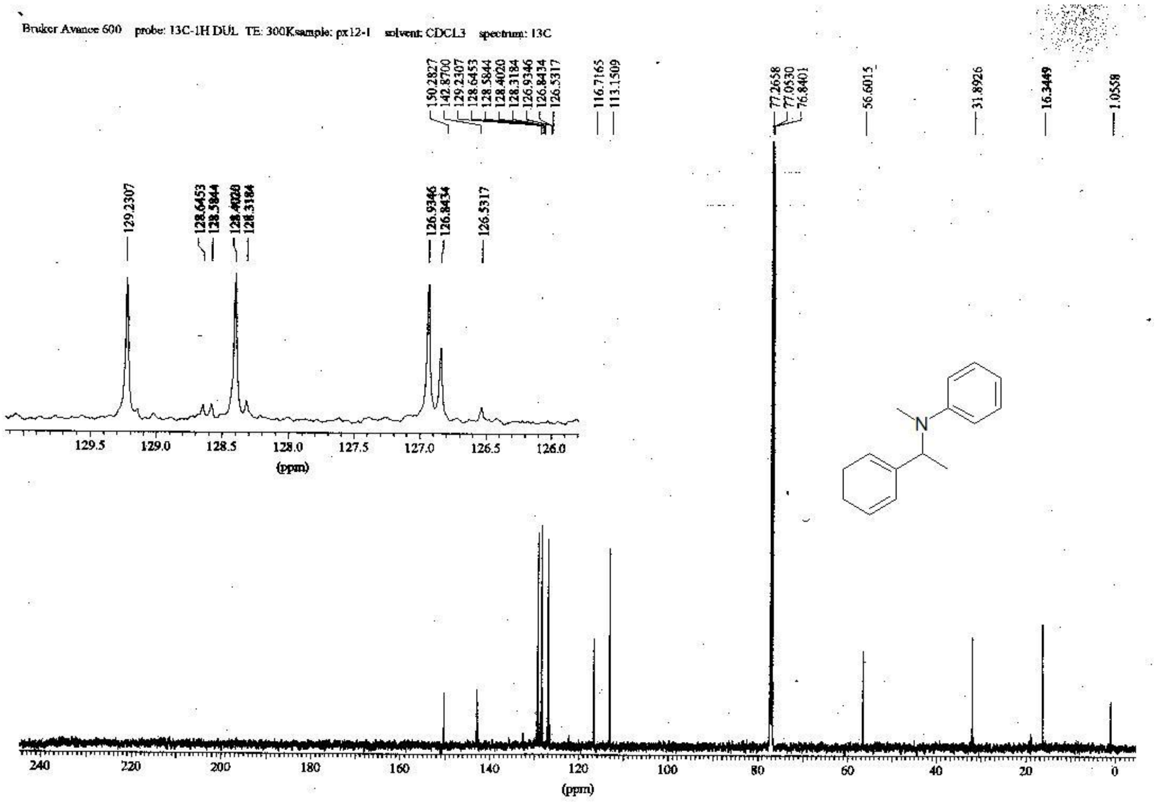

The-NMR spectra of **3b**

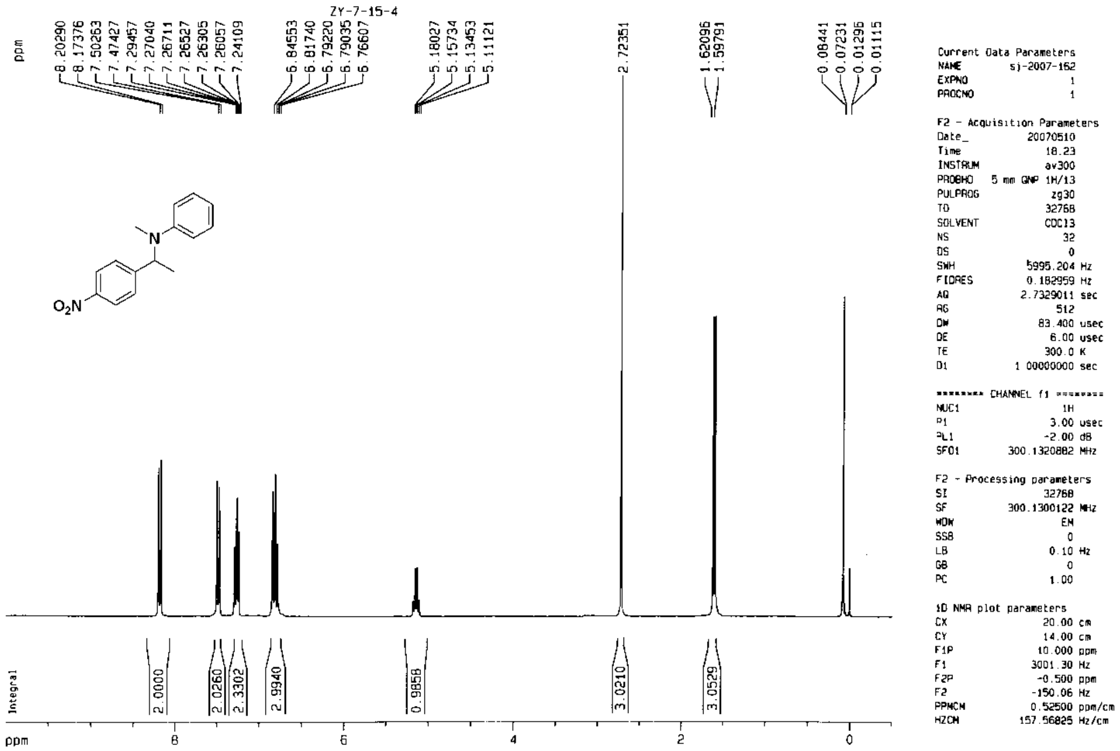

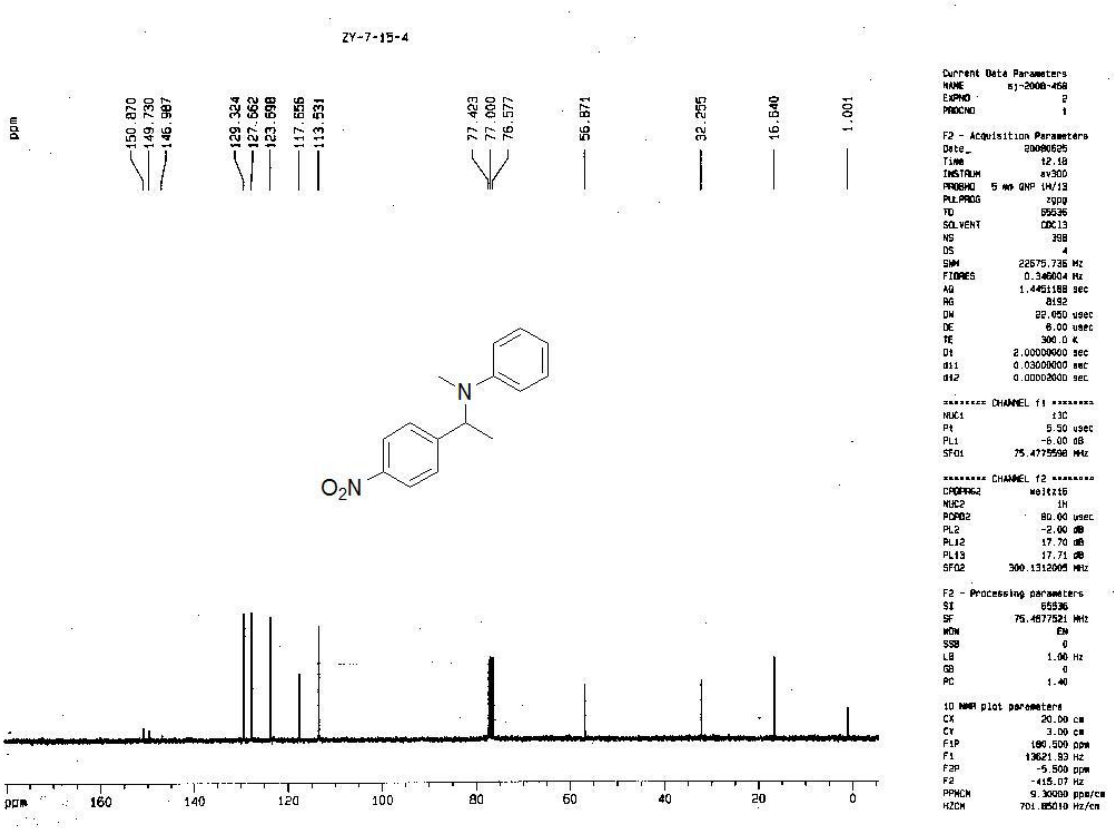

The-NMR spectra of **3c**

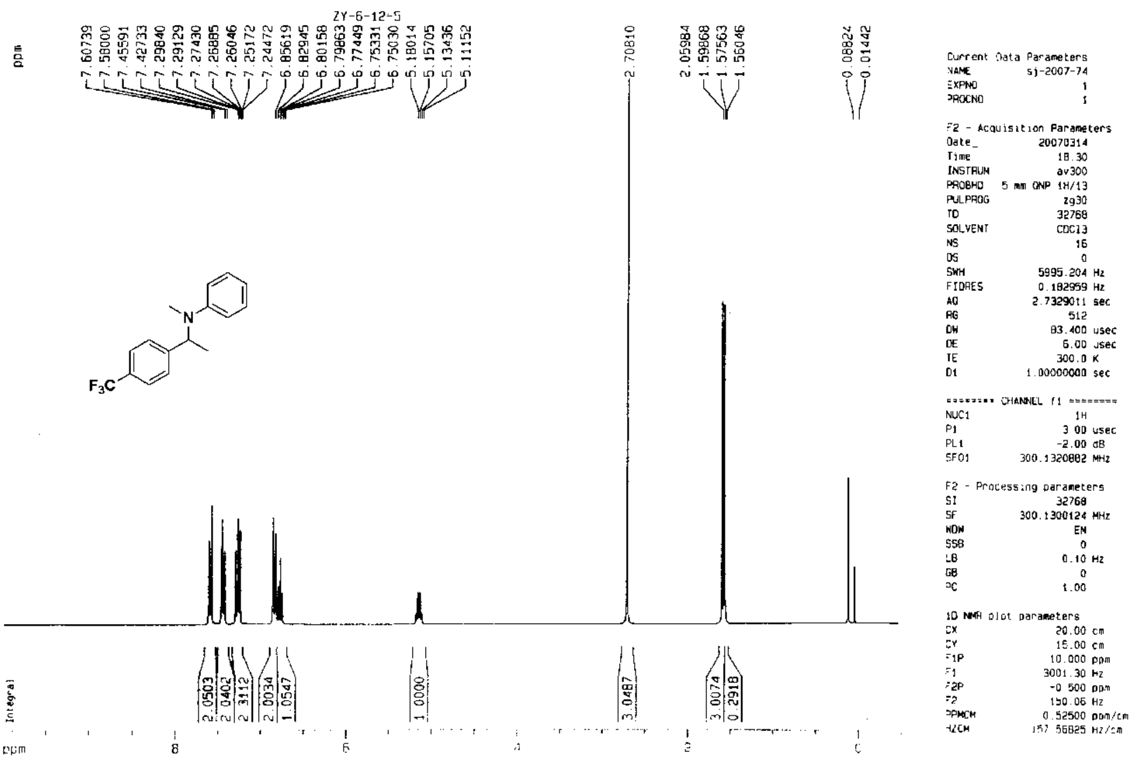

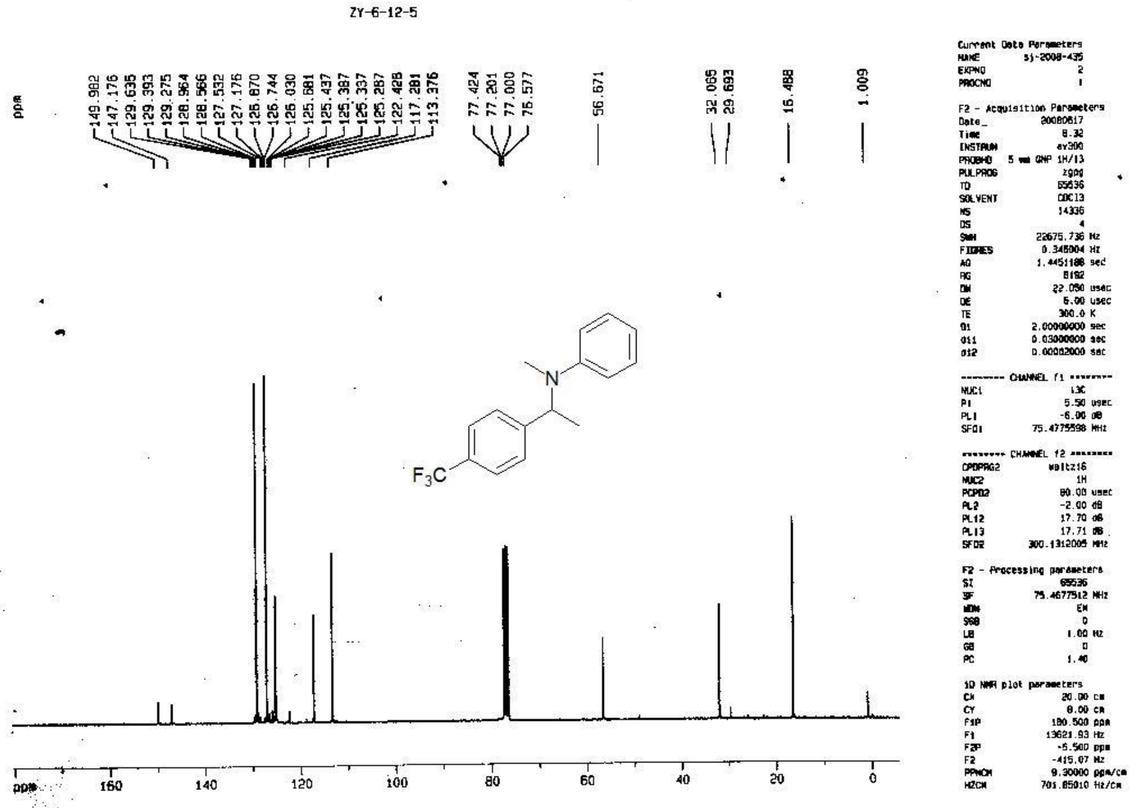

The-NMR spectra of **3d**

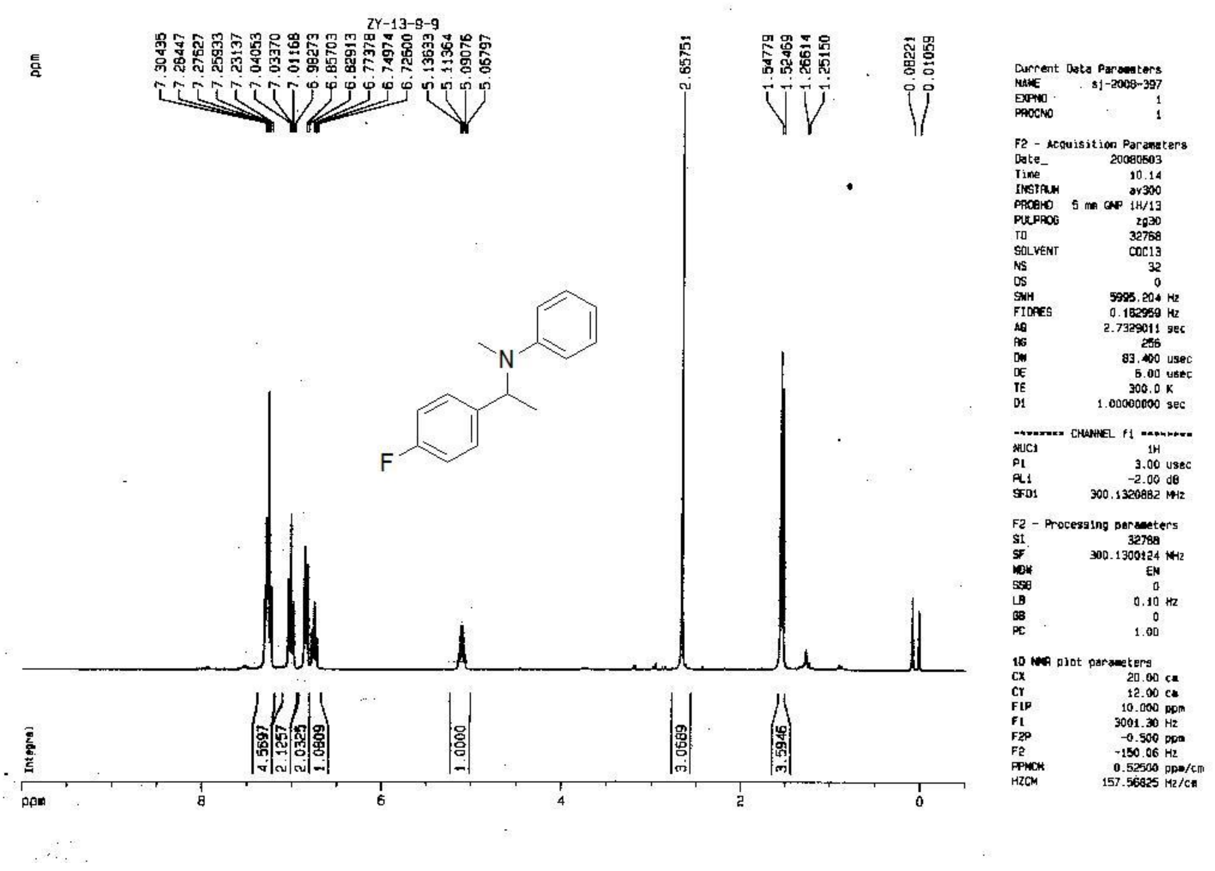

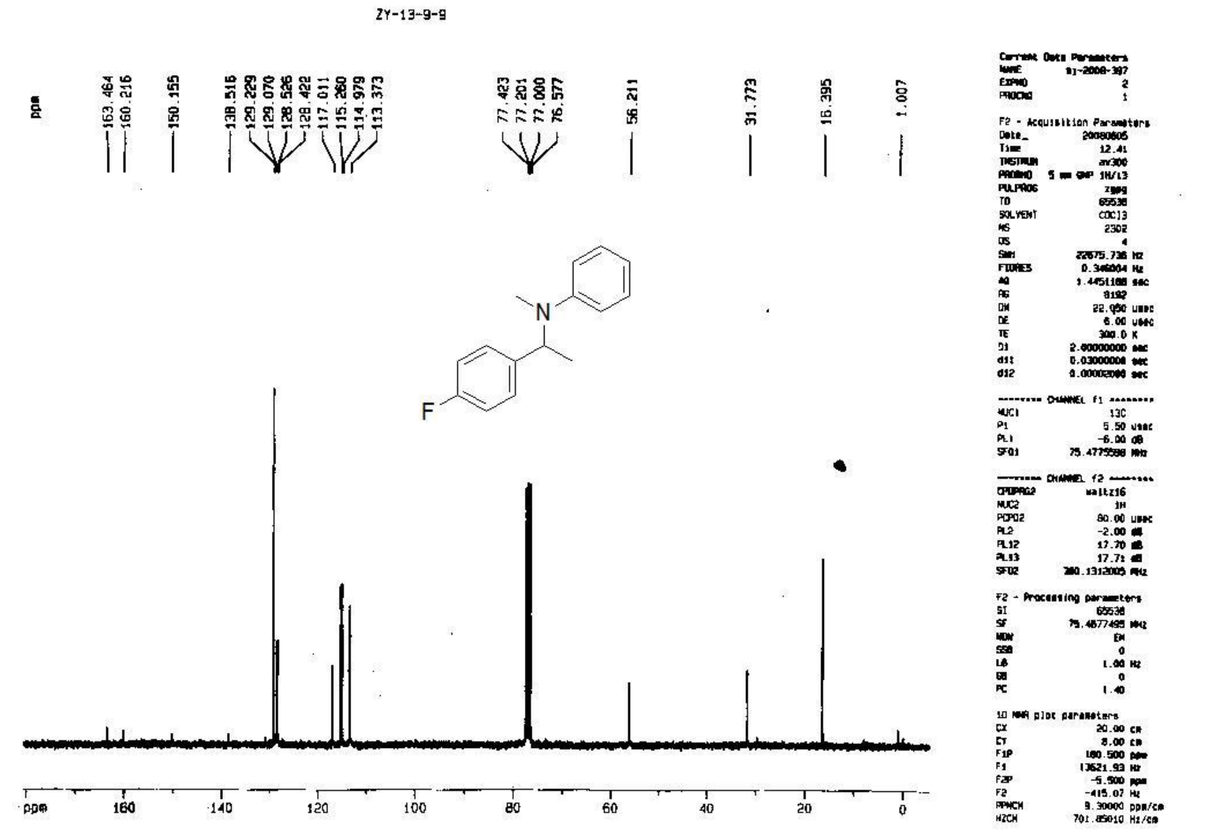

The-NMR spectra of **3e**

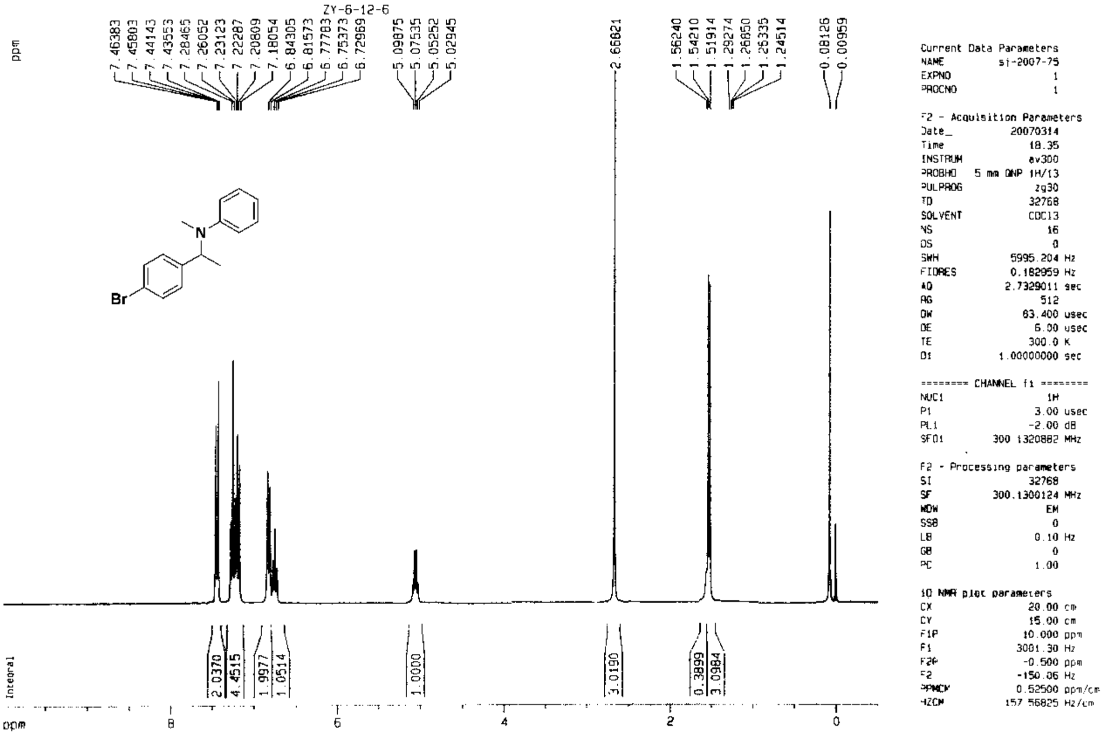

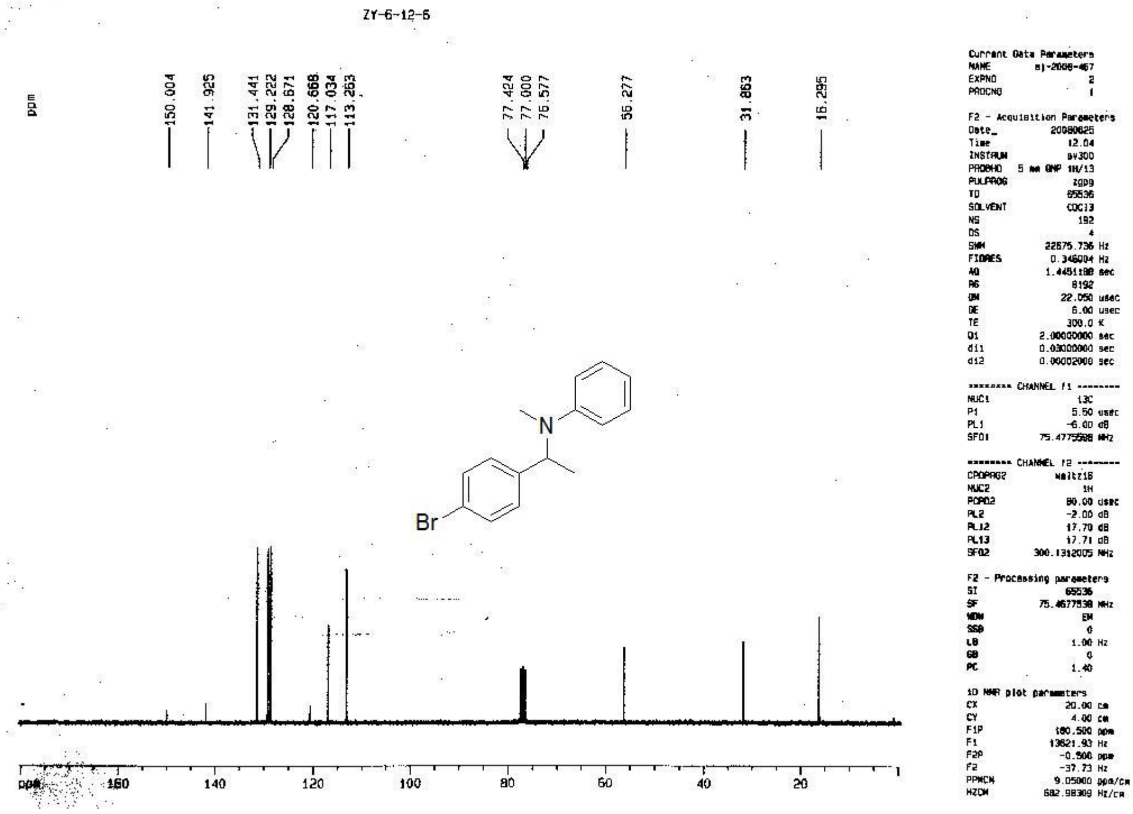

The-NMR spectra of **3f**

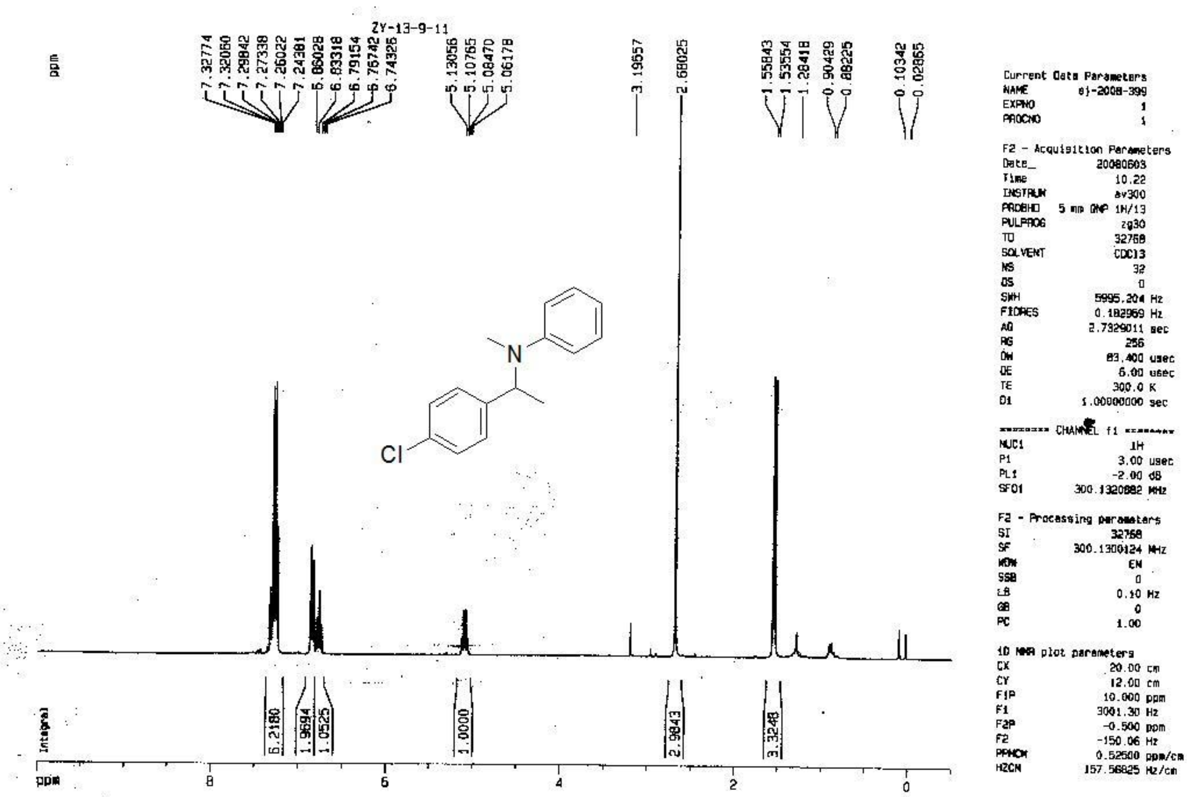

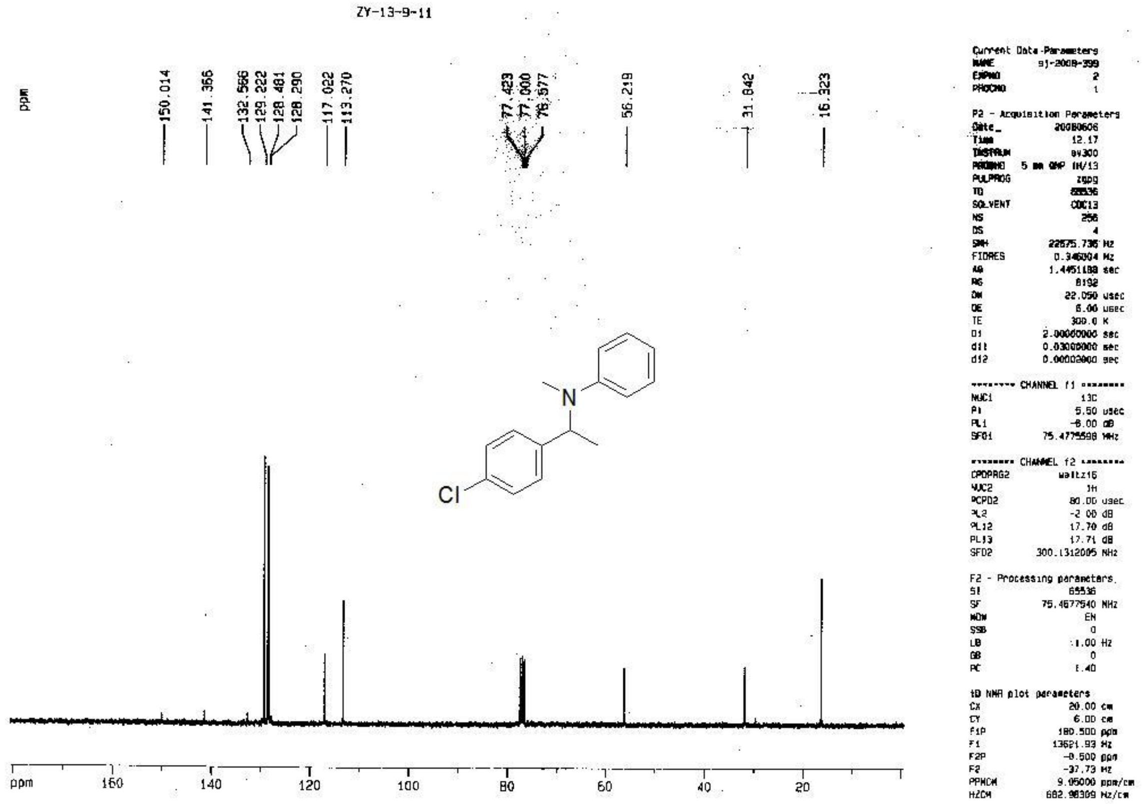

The-NMR spectra of **3g**

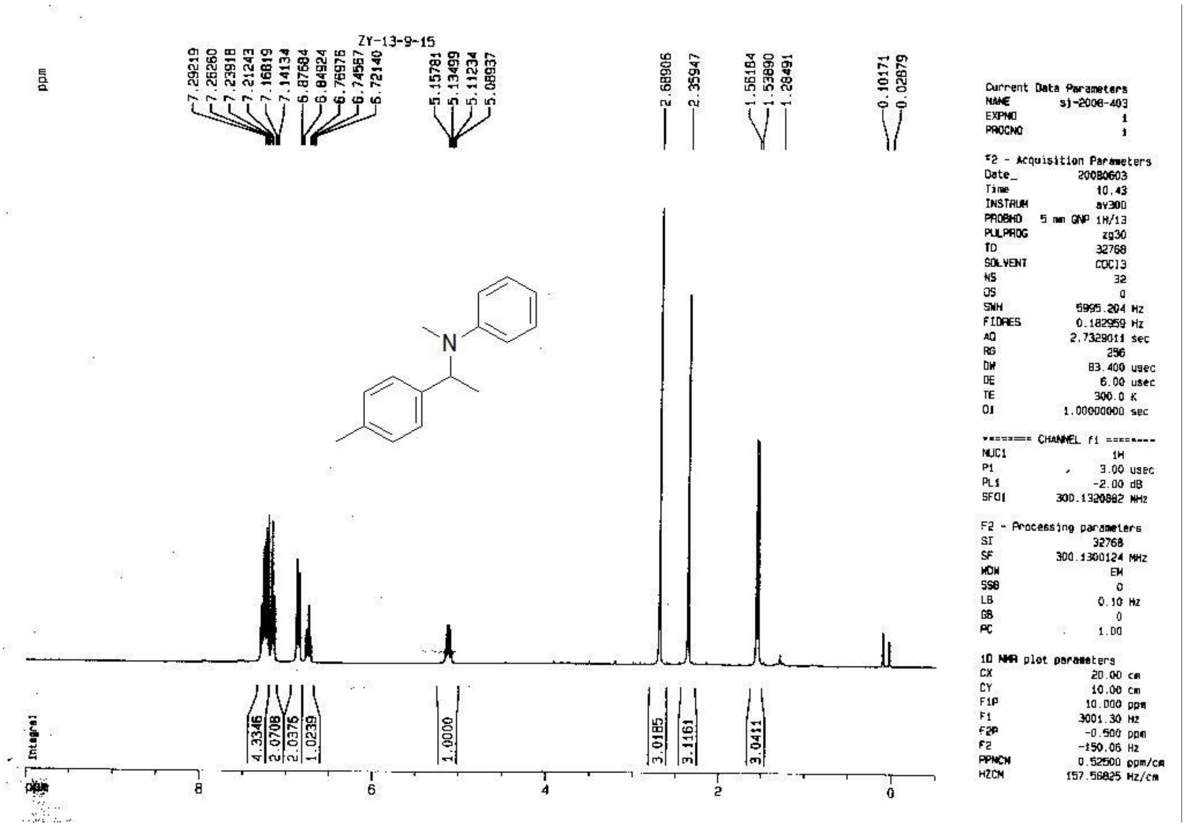

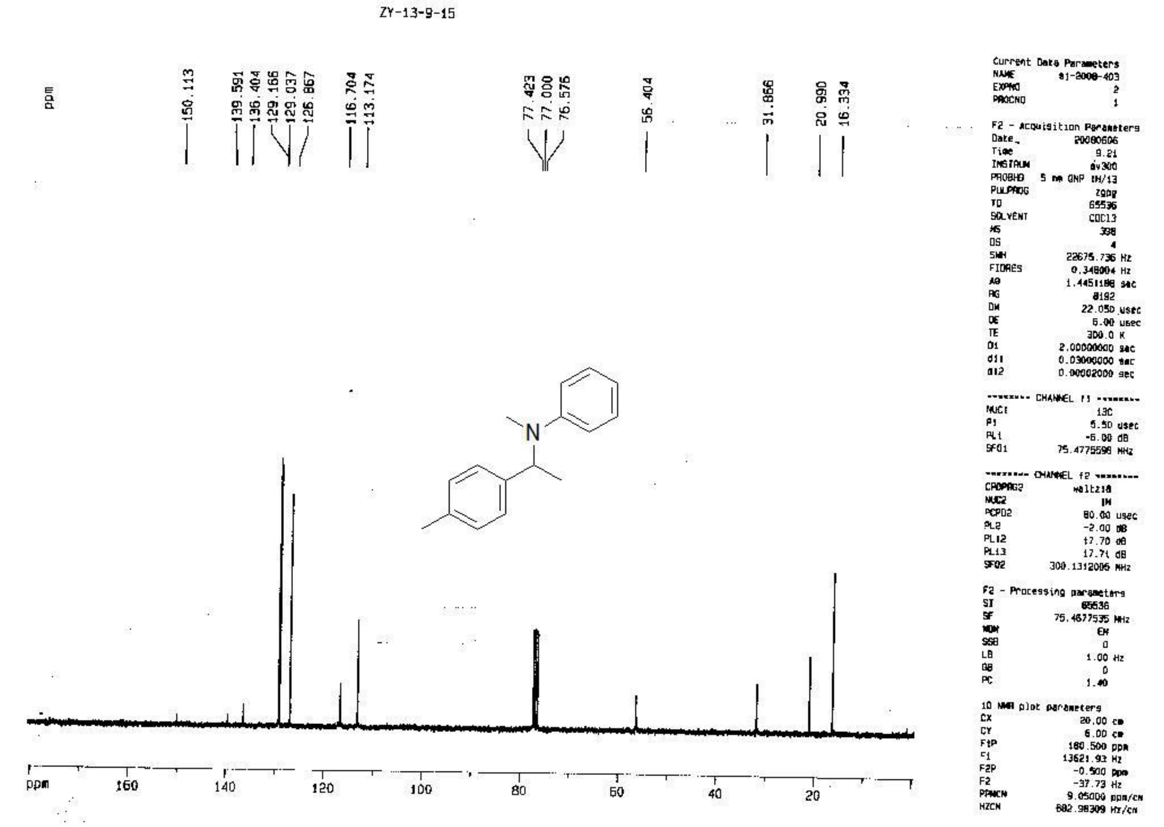

The-NMR spectra of **3h**

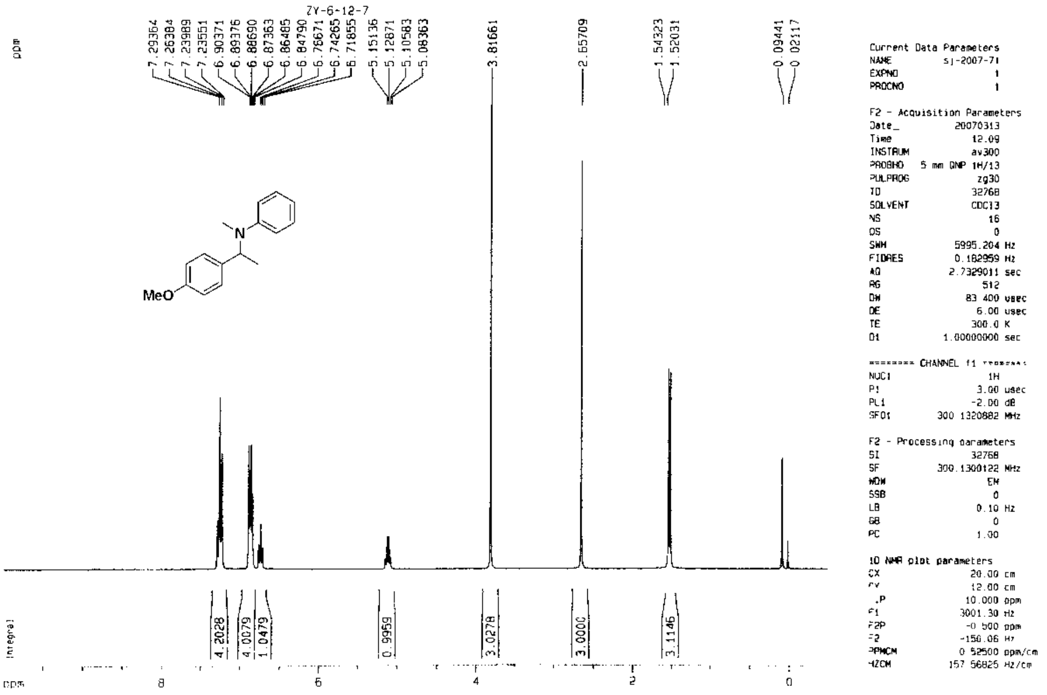

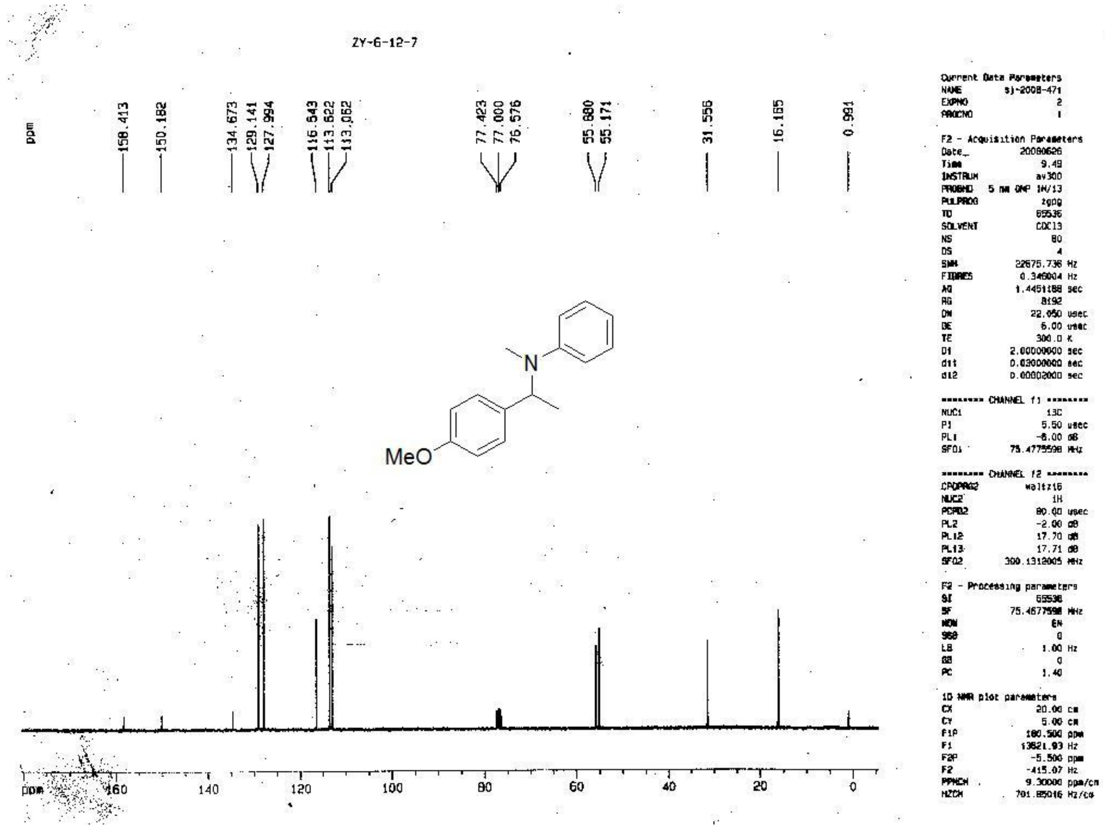

The-NMR spectra of **3i**

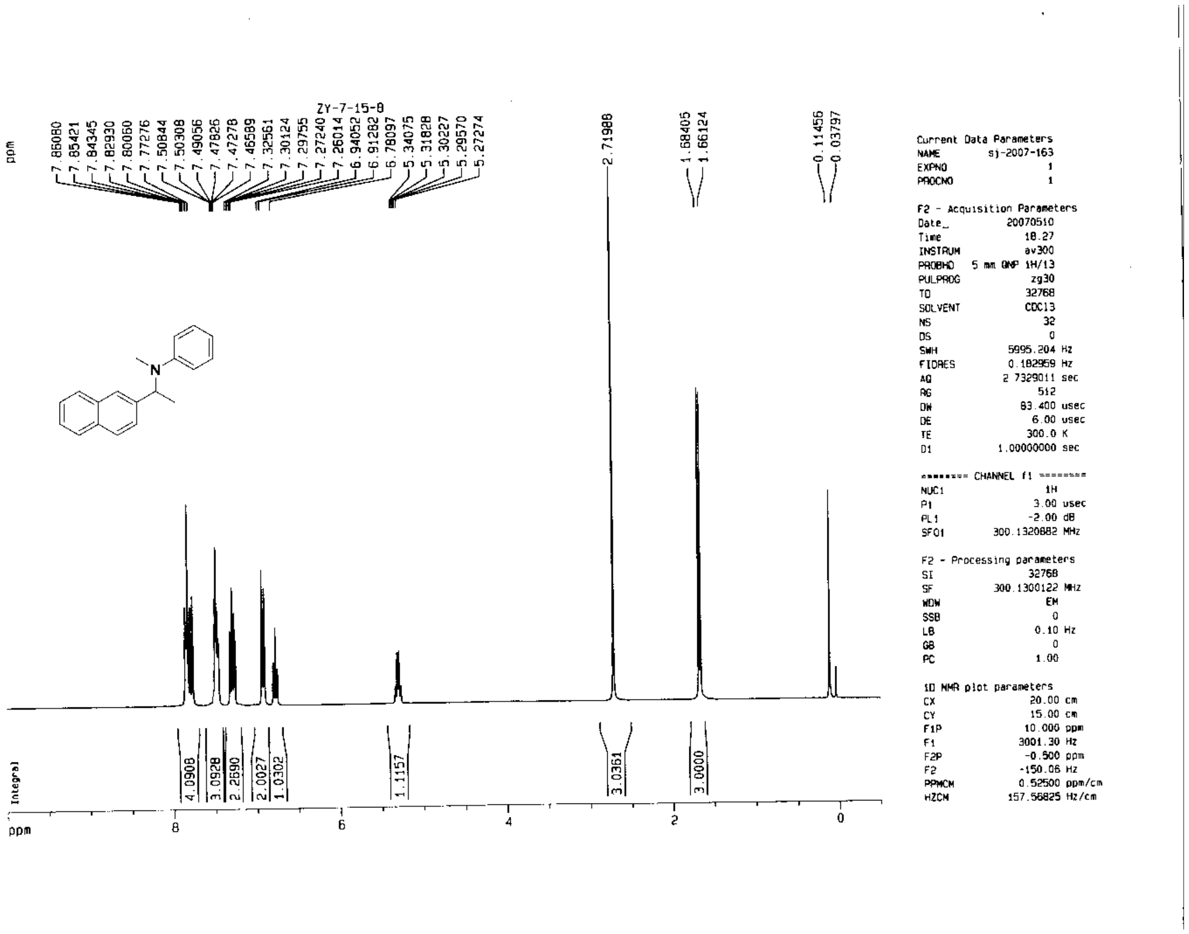

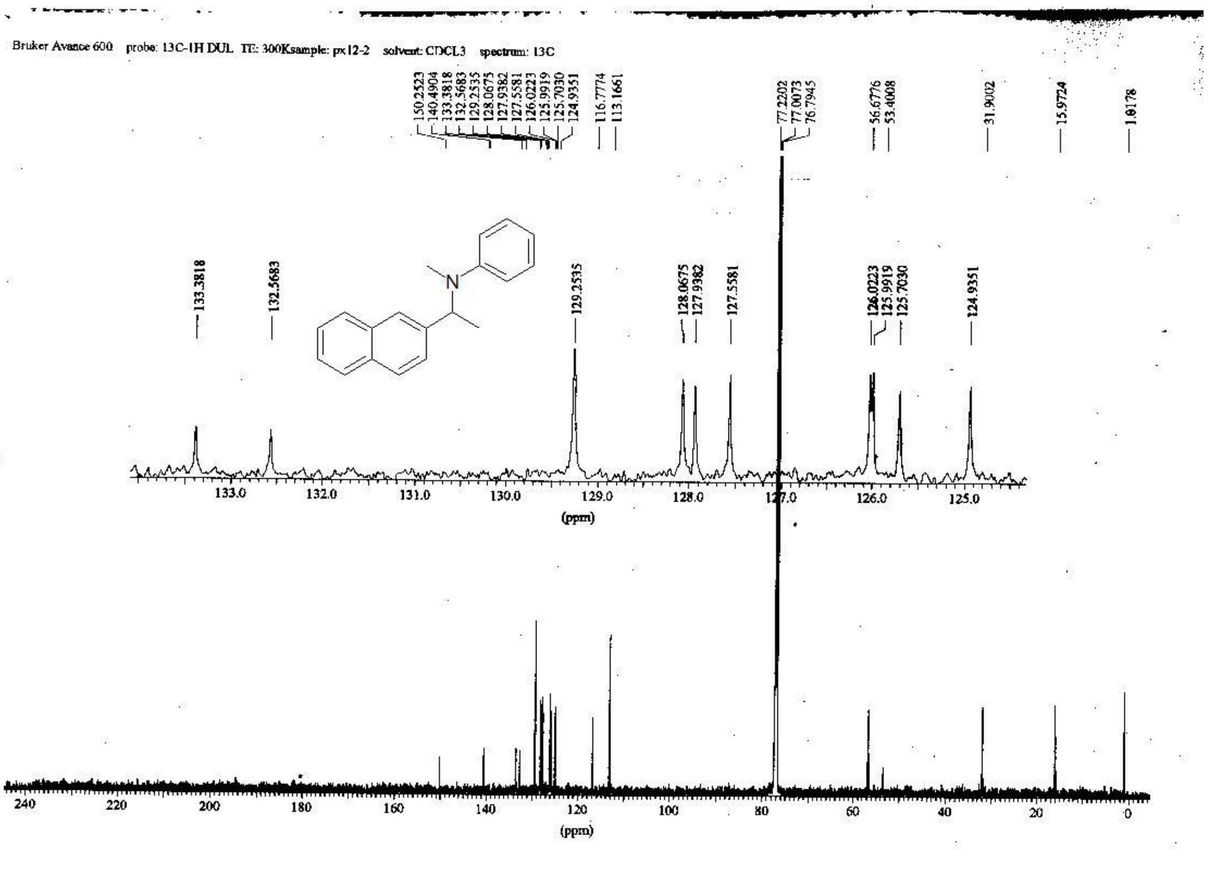

The-NMR spectra of **3j**

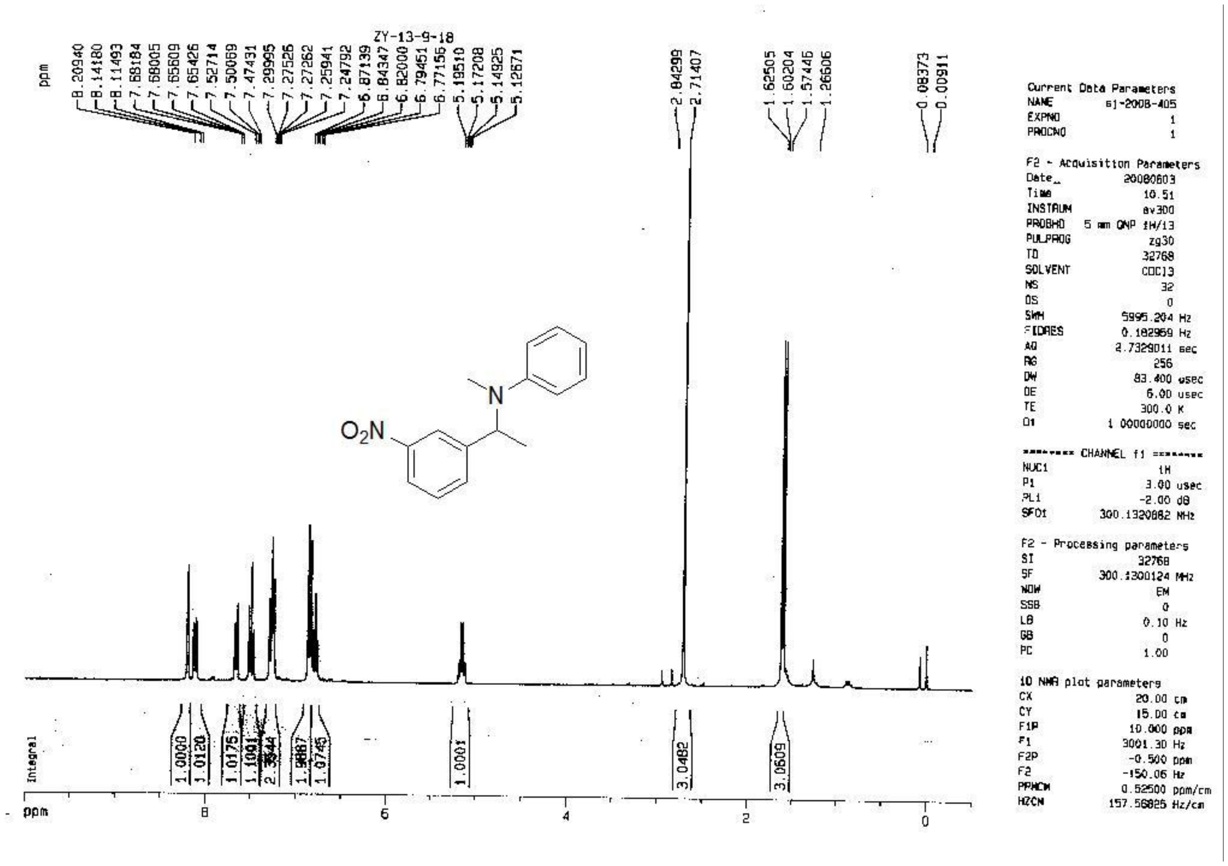

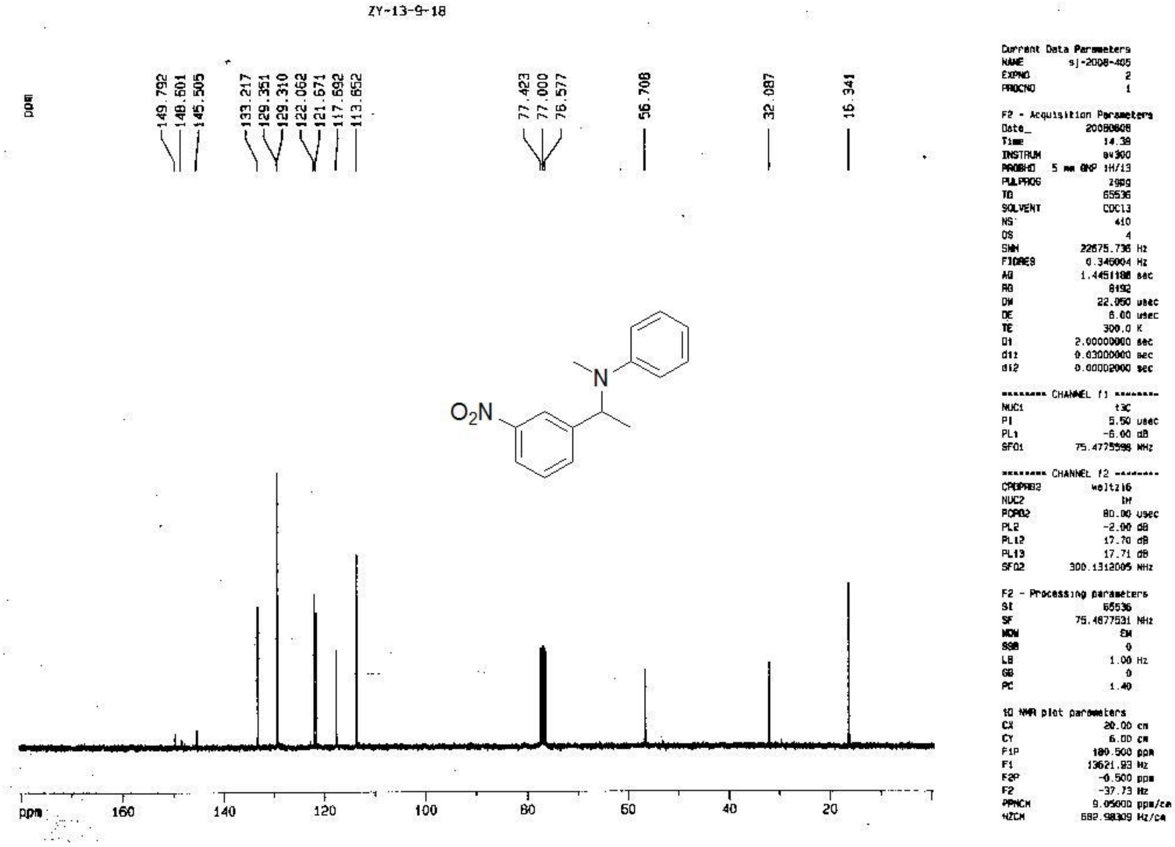

The-NMR spectra of **3k**

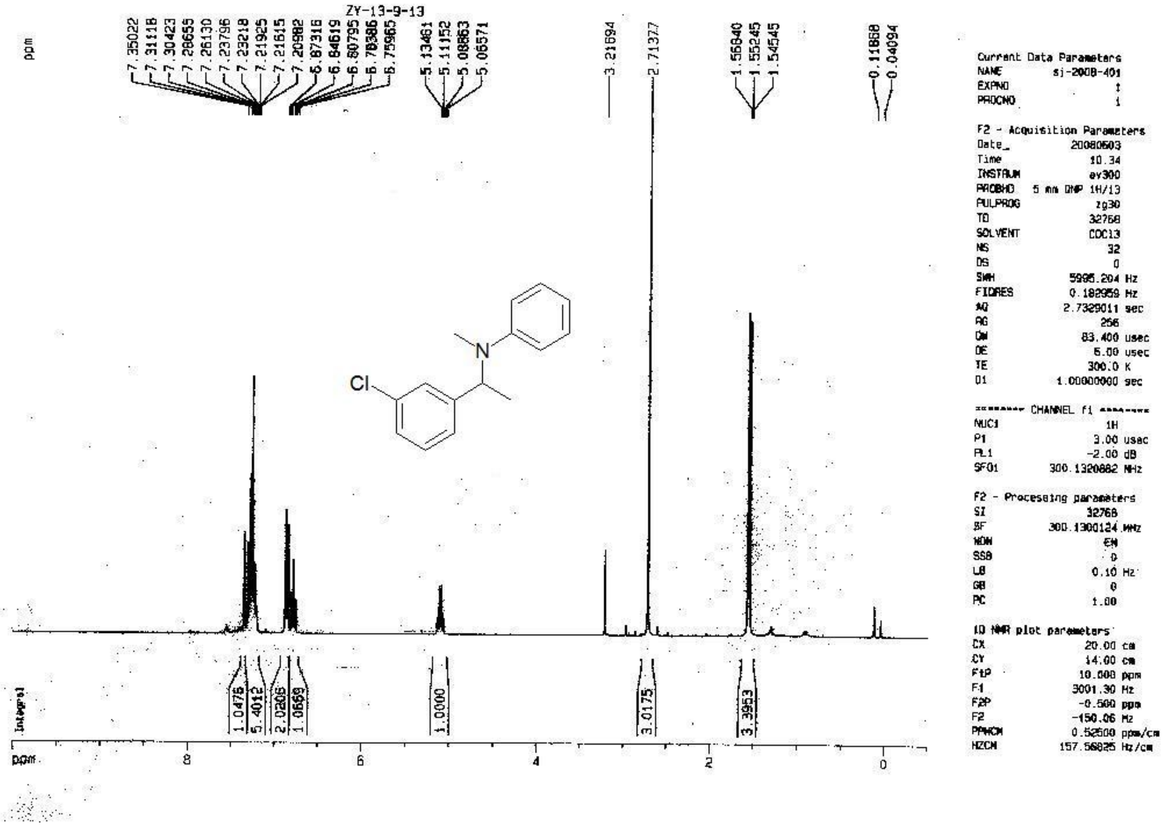

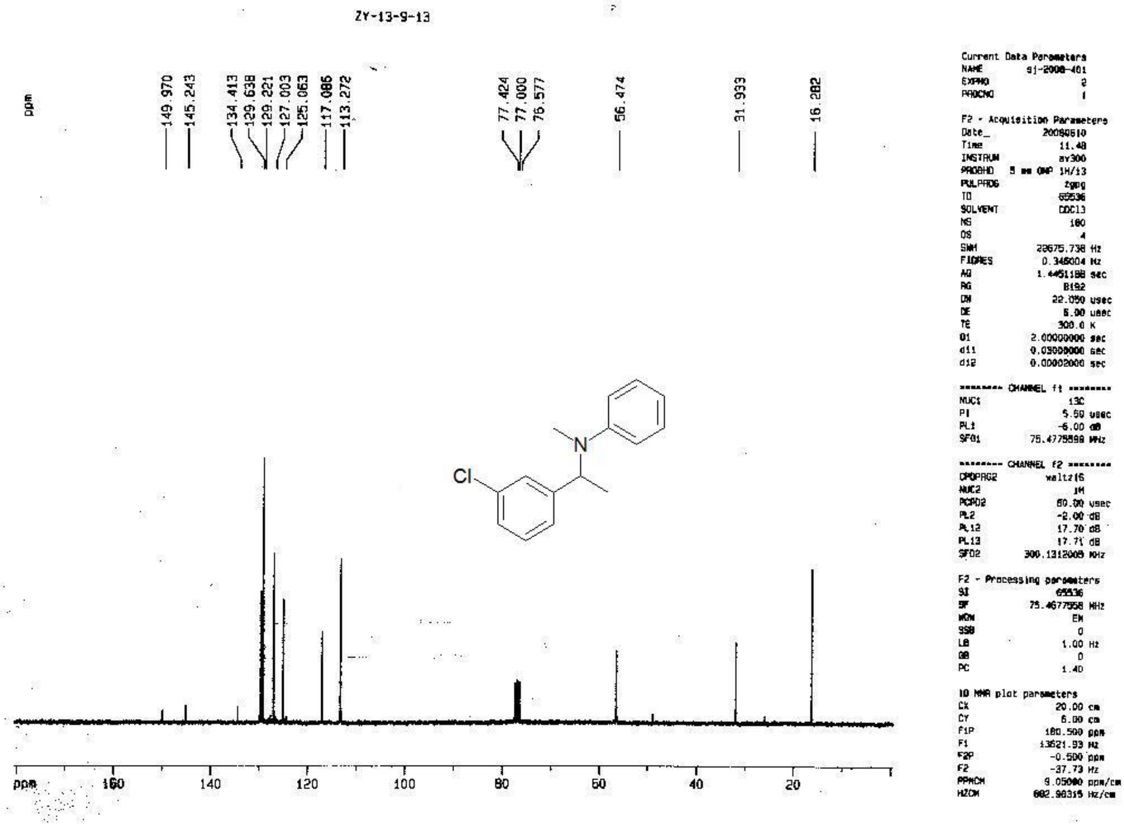

The-NMR spectra of **3l**

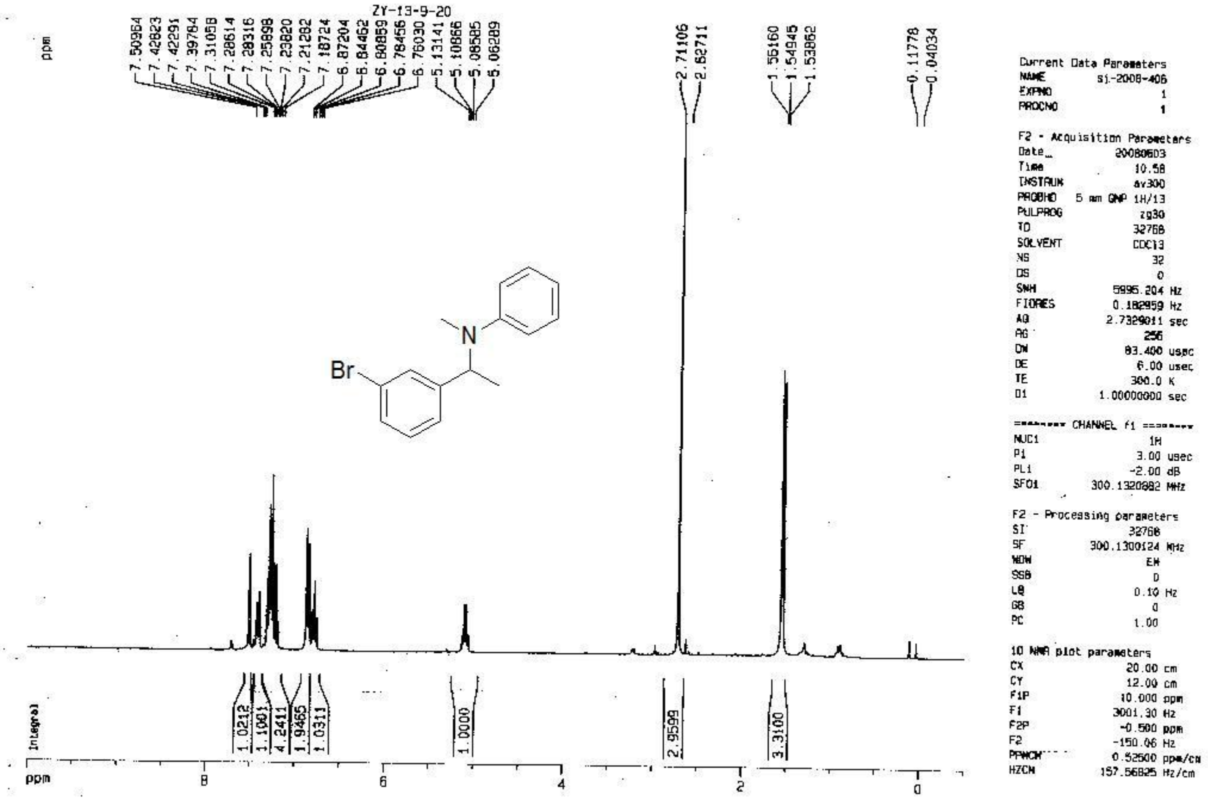

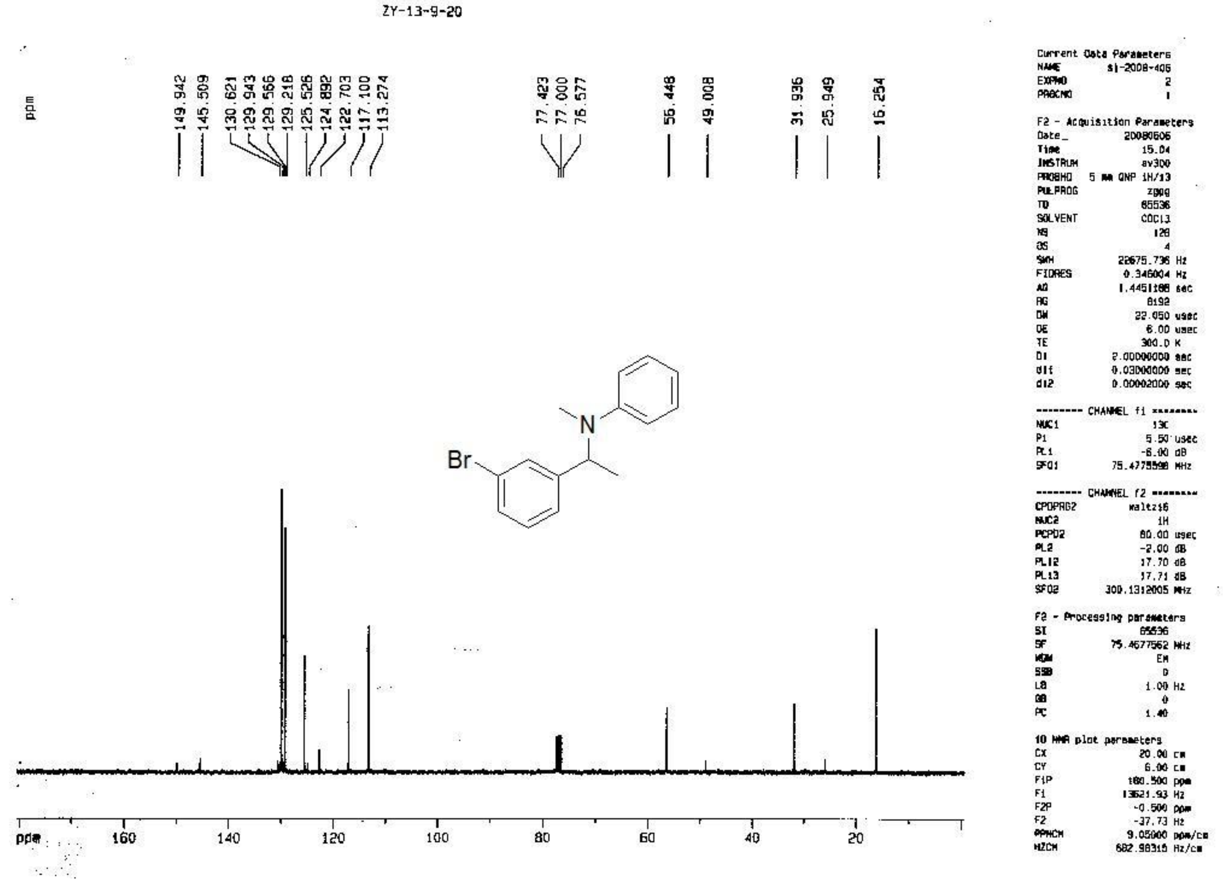

The-NMR spectra of **3m**

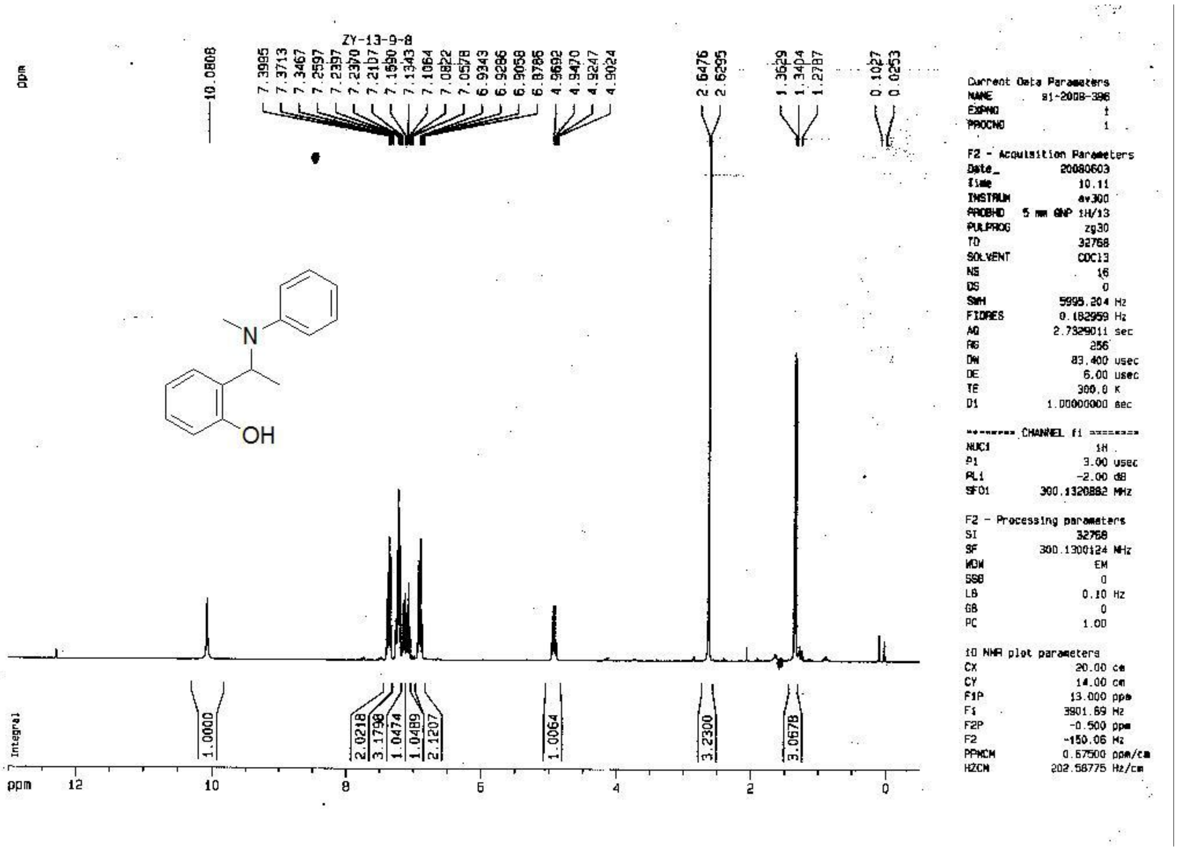

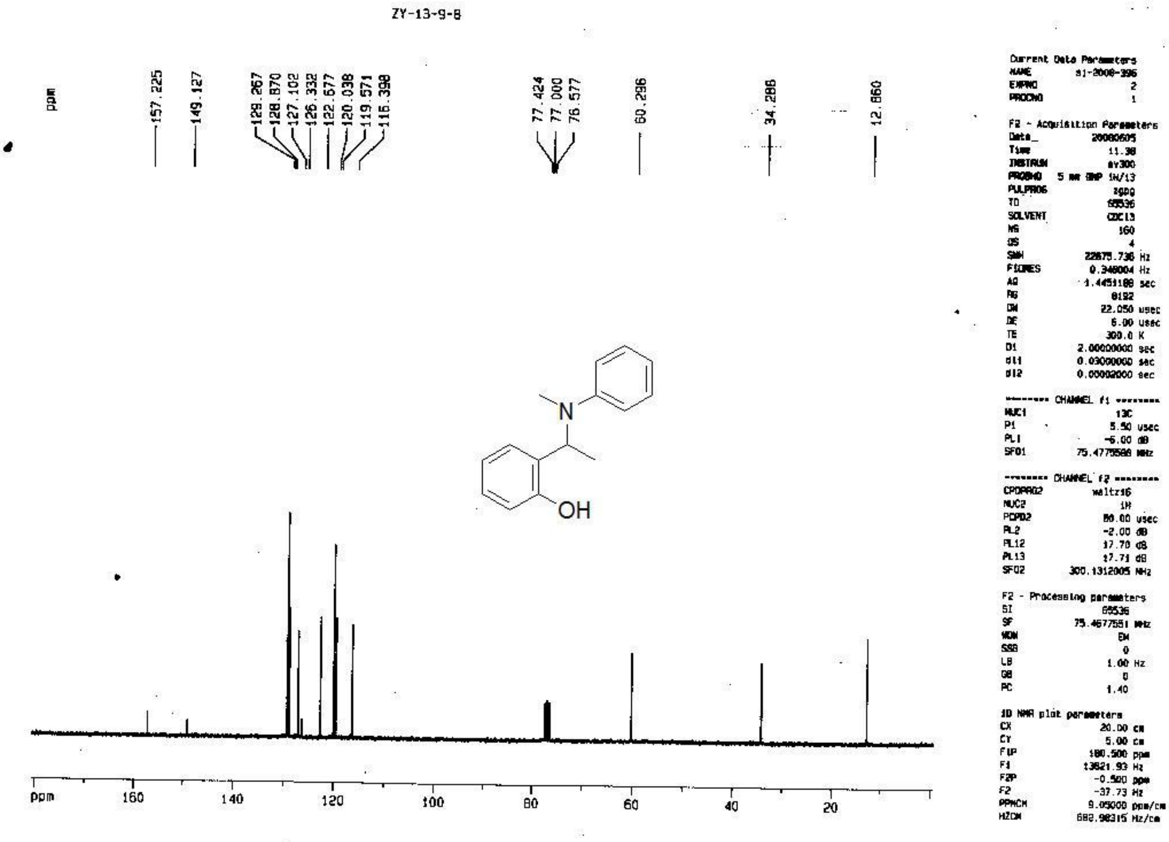

The-NMR spectra of **3n**

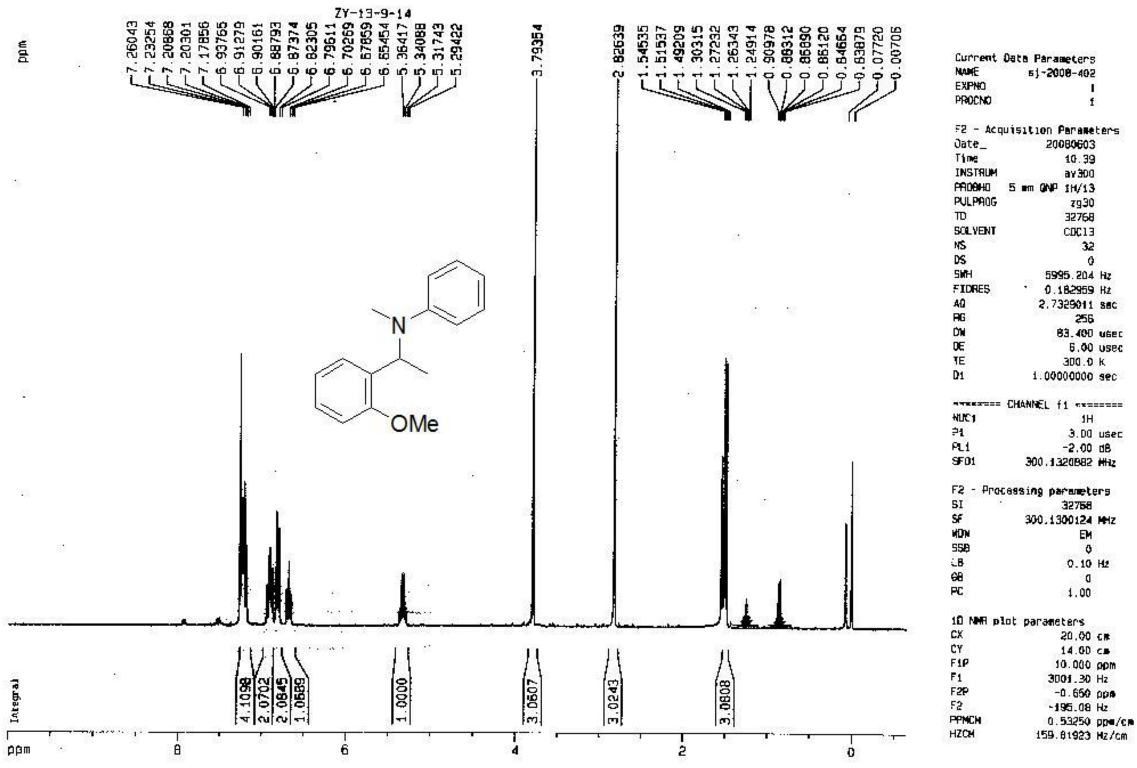

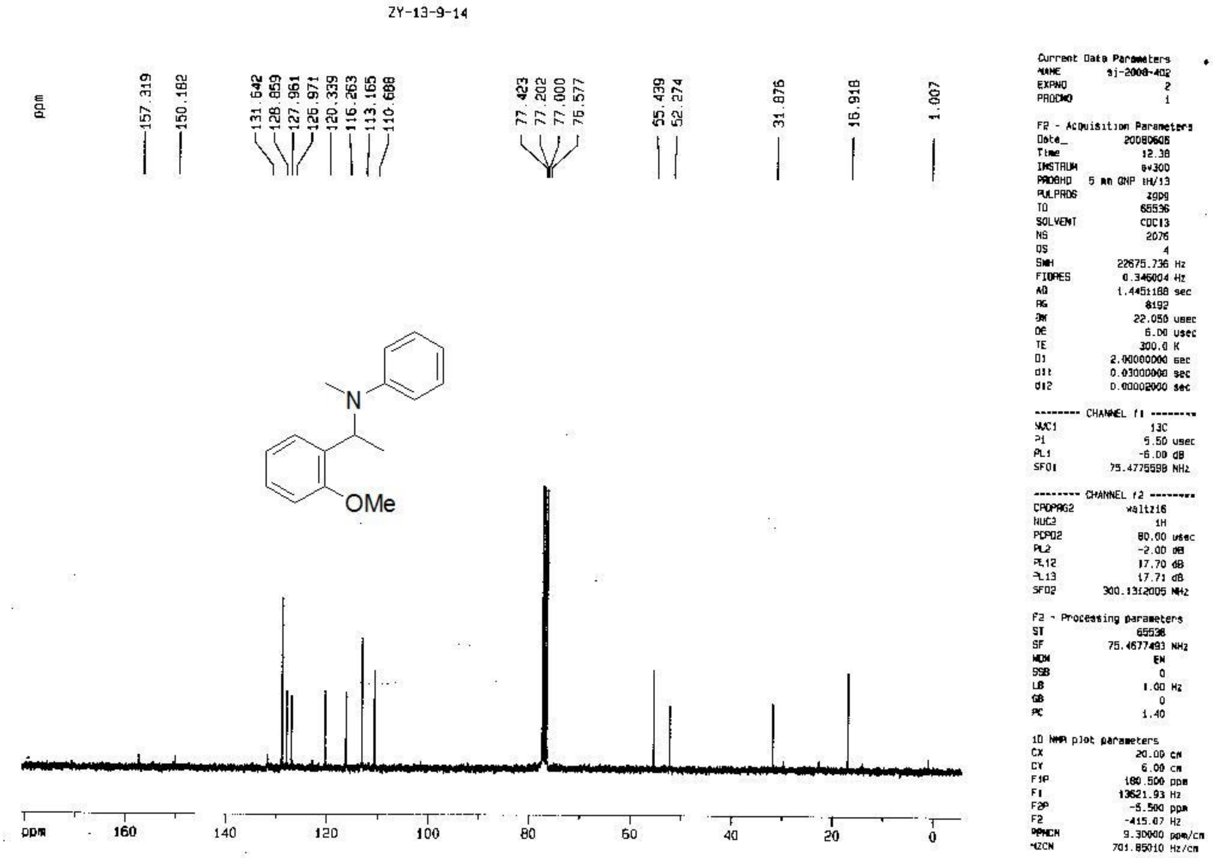

The-NMR spectra of **3o**

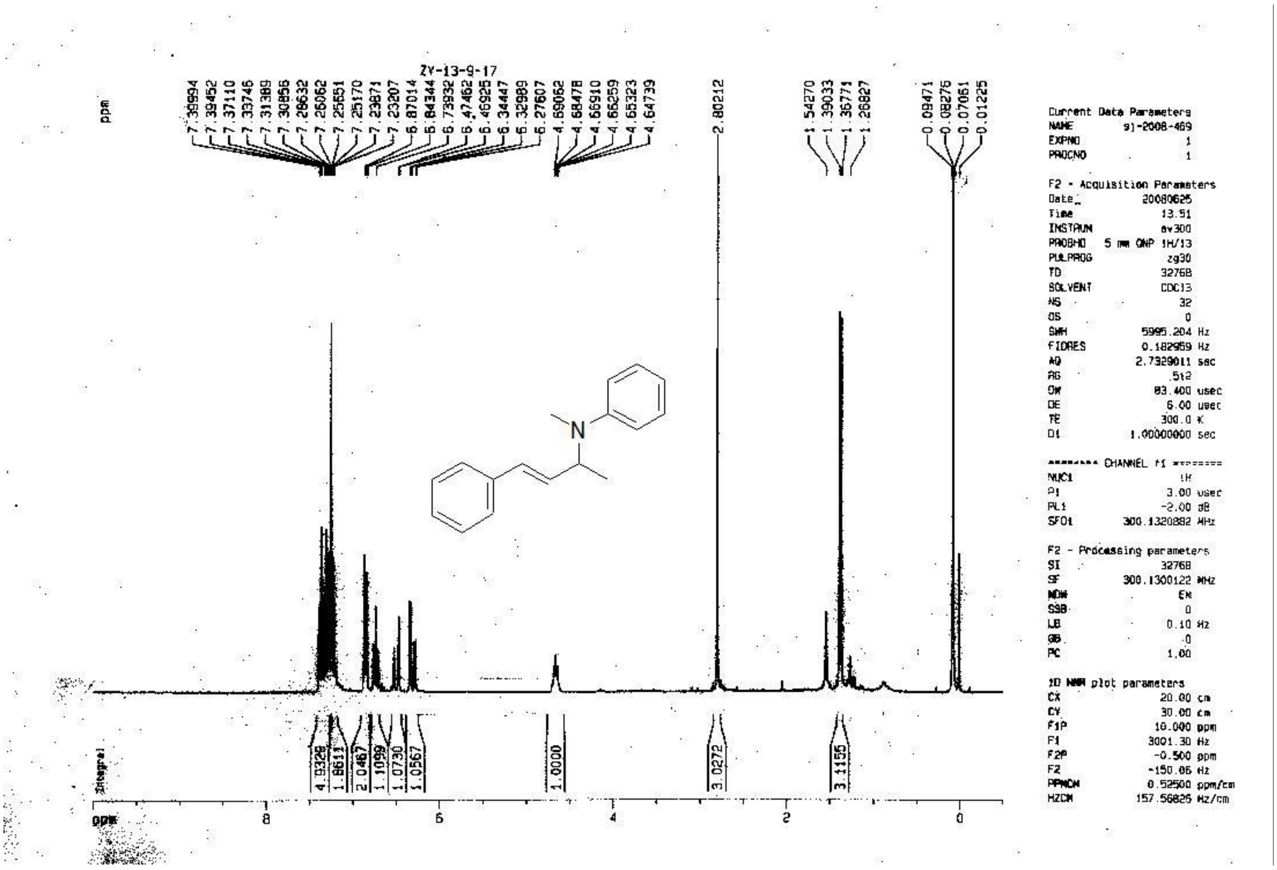

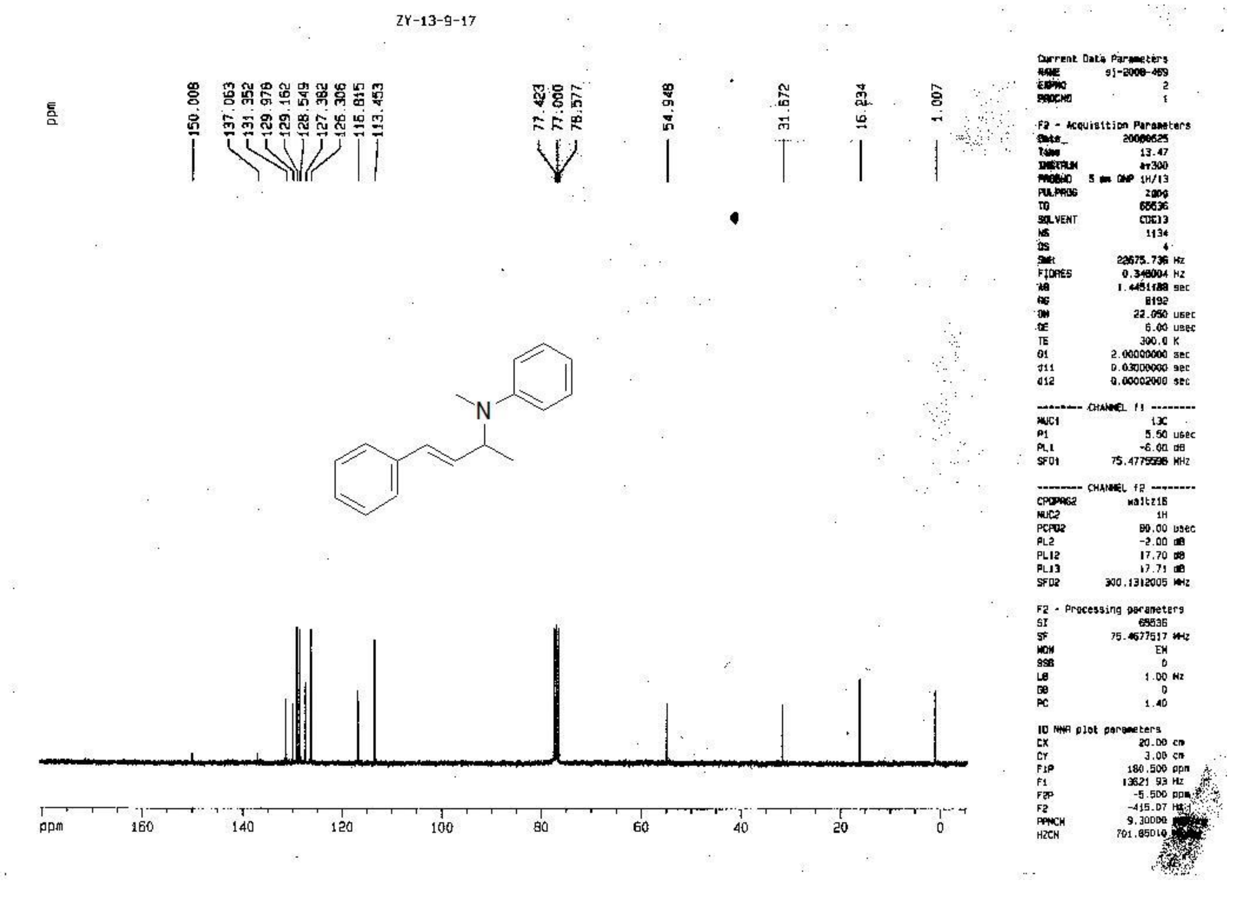

The-NMR spectra of **3p**

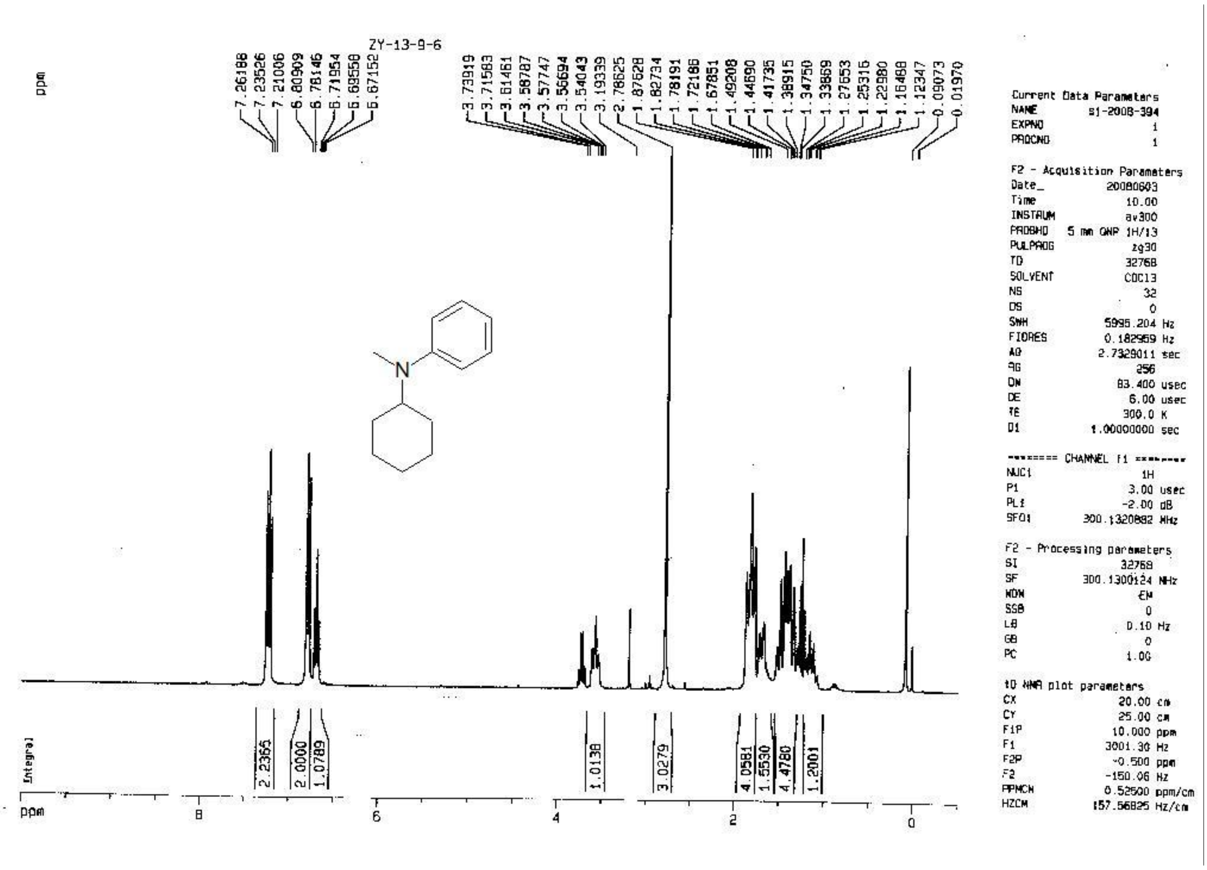

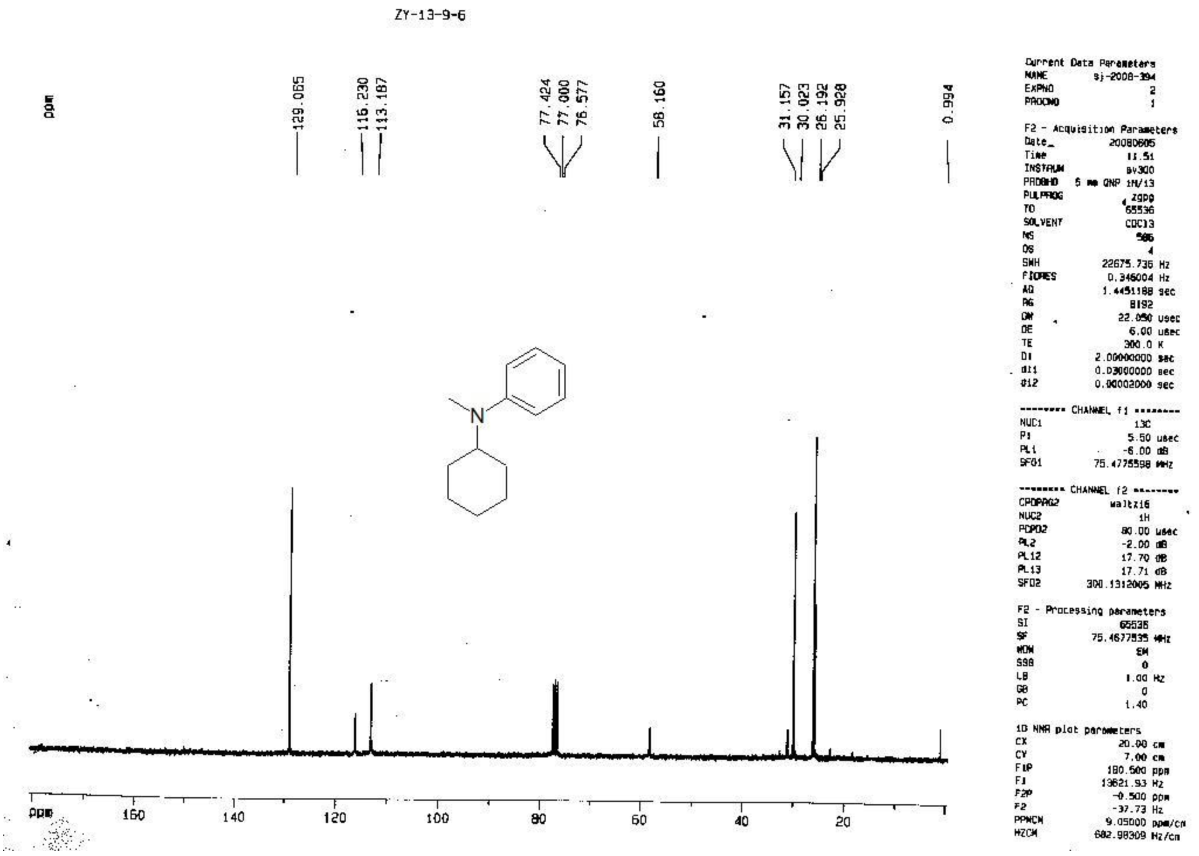

The-NMR spectra of **3q**

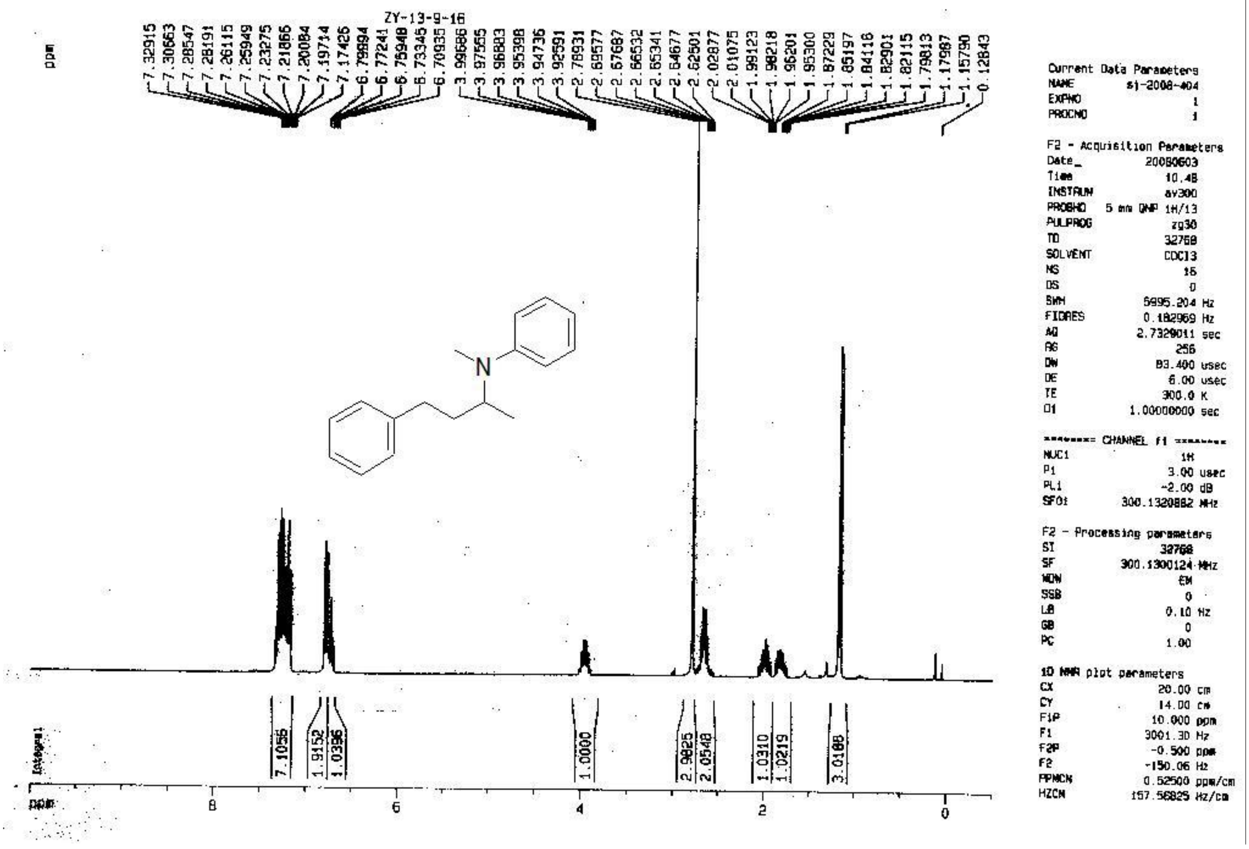

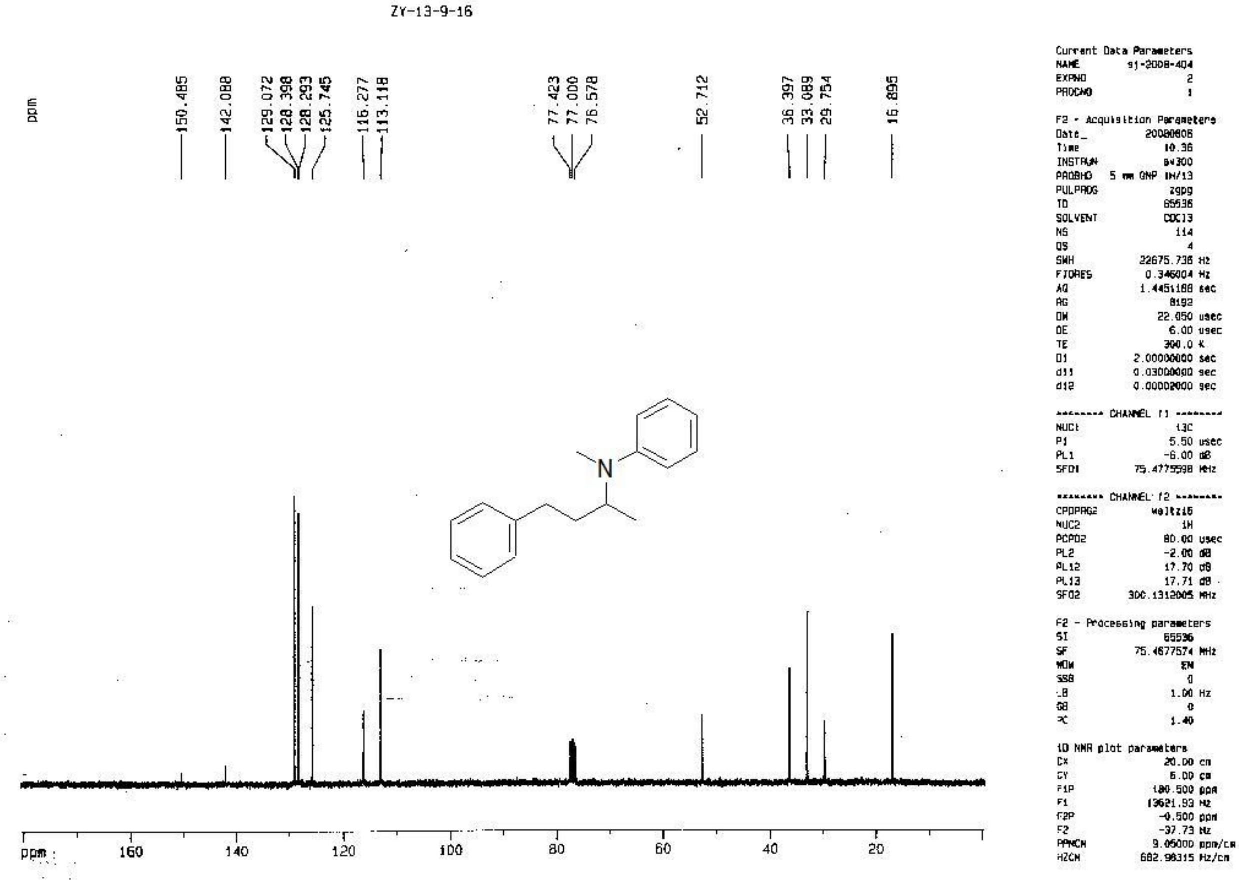

The-NMR spectra of **3r**

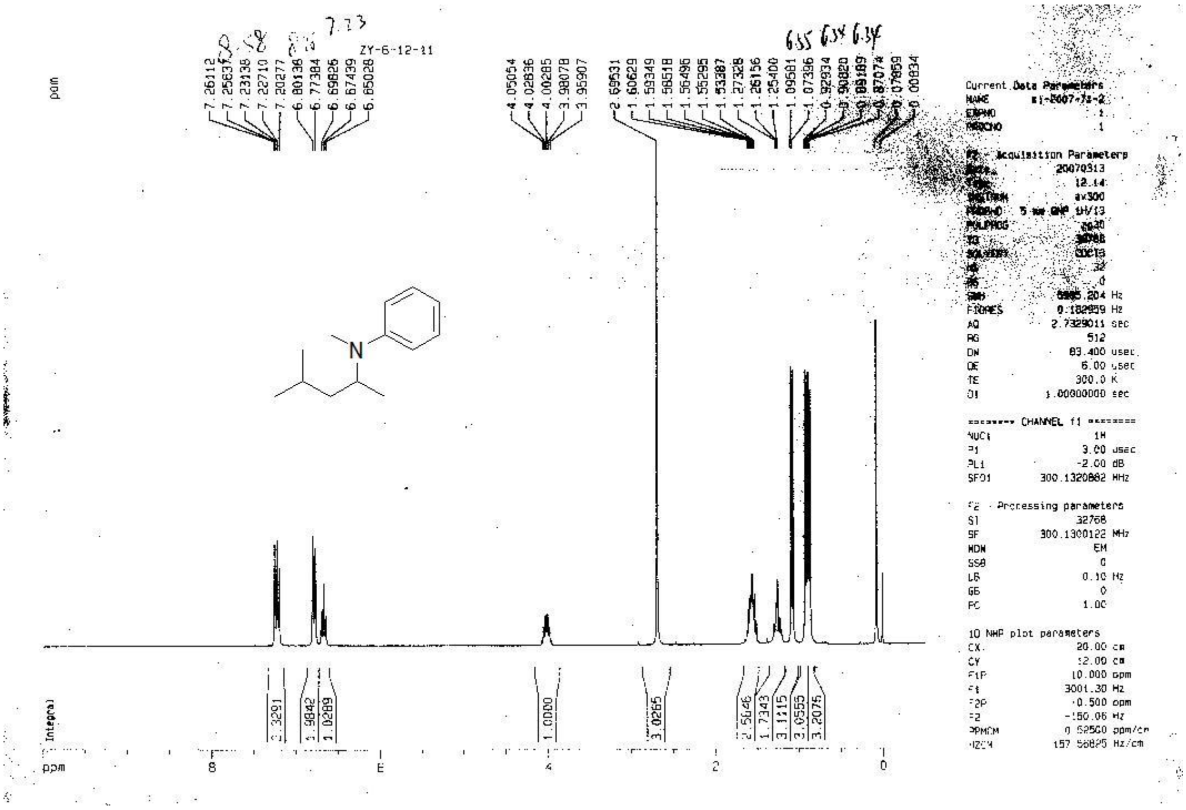

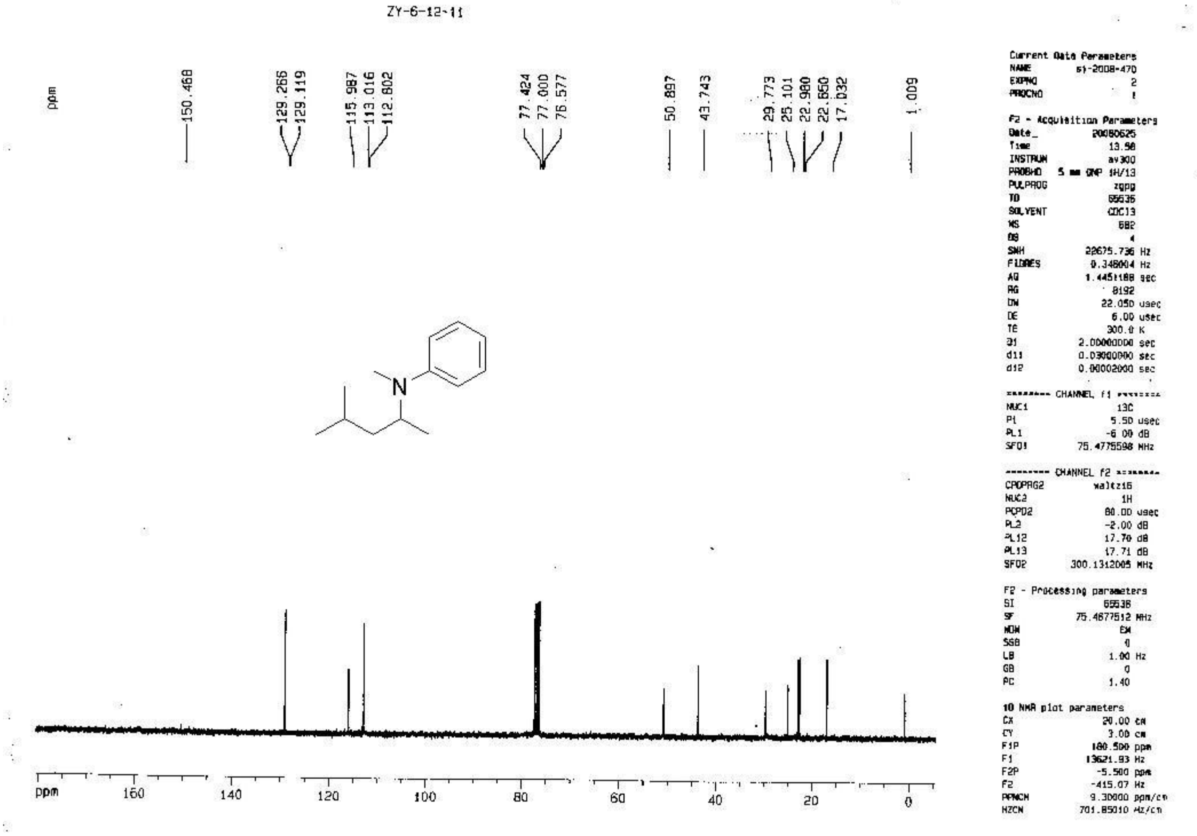

Supplement: Supplementary file 1 [file molecules-17-05151-s001.pdf]
